# Differential gene expression identifies a transcriptional regulatory network involving ER-alpha and PITX1 in invasive epithelial ovarian cancer

**DOI:** 10.1186/s12885-021-08276-8

**Published:** 2021-07-03

**Authors:** Yichao Li, Sushil K. Jaiswal, Rupleen Kaur, Dana Alsaadi, Xiaoyu Liang, Frank Drews, Julie A. DeLoia, Thomas Krivak, Hanna M. Petrykowska, Valer Gotea, Lonnie Welch, Laura Elnitski

**Affiliations:** 1 School of Electrical Engineering and Computer Science, Ohio University, Athens, OH USA; 2 Translational Functional Genomics Branch, National Human Genome Research Institute, National Institutes of Health, Bethesda, MD 20892 USA; 3 Present address: Dignity Health Global Education, Roanoke, Virginia USA; 4 Department of Obstetrics, Gynecology and Reproductive Sciences, University of Pittsburgh Medical School, Pittsburgh, PA USA; 5 Present address: The Western Pennsylvania Hospital, Pittsburgh, PA USA

**Keywords:** High-grade serous ovarian cancer, HGSOC, Serous borderline tumor, SBT, PITX1, Estrogen receptor-alpha, ERα, MegaTrans complex, Low-grade serous ovarian cancer, LGSOC

## Abstract

**Background:**

The heterogeneous subtypes and stages of epithelial ovarian cancer (EOC) differ in their biological features, invasiveness, and response to chemotherapy, but the transcriptional regulators causing their differences remain nebulous.

**Methods:**

In this study, we compared high-grade serous ovarian cancers (HGSOCs) to low malignant potential or serous borderline tumors (SBTs). Our aim was to discover new regulatory factors causing distinct biological properties of HGSOCs and SBTs.

**Results:**

In a discovery dataset, we identified 11 differentially expressed genes (DEGs) between SBTs and HGSOCs. Their expression correctly classified 95% of 267 validation samples. Two of the DEGs, *TMEM30B* and *TSPAN1*, were significantly associated with worse overall survival in patients with HGSOC. We also identified 17 DEGs that distinguished stage II vs. III HGSOC. In these two DEG promoter sets, we identified significant enrichment of predicted transcription factor binding sites, including those of RARA, FOXF1, BHLHE41, and PITX1. Using published ChIP-seq data acquired from multiple non-ovarian cell types, we showed additional regulatory factors, including AP2-gamma/TFAP2C, FOXA1, and BHLHE40, bound at the majority of DEG promoters. Several of the factors are known to cooperate with and predict the presence of nuclear hormone receptor estrogen receptor alpha (ER-alpha). We experimentally confirmed ER-alpha and PITX1 presence at the DEGs by performing ChIP-seq analysis using the ovarian cancer cell line PEO4. Finally, RNA-seq analysis identified recurrent gene fusion events in our EOC tumor set. Some of these fusions were significantly associated with survival in HGSOC patients; however, the fusion genes are not regulated by the transcription factors identified for the DEGs.

**Conclusions:**

These data implicate an estrogen-responsive regulatory network in the differential gene expression between ovarian cancer subtypes and stages, which includes PITX1. Importantly, the transcription factors associated with our DEG promoters are known to form the MegaTrans complex in breast cancer. This is the first study to implicate the MegaTrans complex in contributing to the distinct biological trajectories of malignant and indolent ovarian cancer subtypes.

**Supplementary Information:**

The online version contains supplementary material available at 10.1186/s12885-021-08276-8.

## Introduction

Epithelial ovarian cancer (EOC) accounts for over 90% of ovarian cancer cases [1] and results in 14,000 deaths in the United States every year, making it the leading cause of death attributed to gynecological cancers [2]. Current scientific advances provide hope that new treatments—immunotherapies, PARP inhibitors, and small molecules tailored to tumor characteristics—can improve patient survival [3]. However, these new treatments have not yet increased the 5-year survival rate for late-stage, invasive EOC, which has been near 30% for several decades [4]. One impediment to treating EOC is the heterogeneity of the disease, which is complicated by multiple histopathological subtypes that differ in morphology, prognosis, etiology, mutational landscape, and chemotherapeutic response [5].

Serous ovarian cancers are the most common type of EOC. They are divided into two groups, according to their invasiveness and aggressiveness: high-grade serous ovarian cancer (HGSOC) and low-grade serous ovarian cancer (LGSOC) [6]. HGSOCs comprise 70% of all EOCs, initially respond to chemotherapy, subsequently relapse, and predict overall survival (OS) of 54–57 months [7]. LGSOCs comprise 4–10% of EOCs, are less responsive to chemotherapy, and predict a longer OS, of 82–126 months [7, 8]. Both HGSOCs and LGSOCs are invasive and can progress to late-stage, untreatable disease; whereas 5-year survival rates favor LGSOCs (62.3% vs. 43.9% for HGSOCs [8]), 10-year survival rates for HGSOCs and LGSOCs are not statistically different (21.2% vs. 22.7%, respectively; *P* = 0.17 [8]). The two EOC subtypes differ in their oncogenic pathways, which are driven by distinct mutational profiles that contain either *TP53* (HGSOCs) or *BRAF*, *KRAS,* and *NRAS* mutations (LGSOCs) [9]. For the purposes of comparison, HGSOCs and LGSOCs each have invasive properties, and the standard of care is identical for late-stage disease of either type: maximal surgical debulking followed by combination chemotherapy with platinum and taxane doublets [10]. Whereas HGSOCs are initially chemosensitive, LGSOCs are typically less chemosensitive. Additionally, due to the difference in molecular characteristics in LGSOC, utilization of hormonal agents as therapy in these patients are currently ongoing.

Many published EOC studies include HGSOCs but not LGSOCs, because of the rarity of the latter. Thus, the molecular foundations underlying the different characteristics of these subtypes are not completely known. Beyond driver mutations, known differences between the subtypes include, but are not limited to, proteins involved in the epithelial to mesenchymal transition [11], MAP kinase signaling [12], metalloproteinases and their inhibitors [13], and homologous recombination deficiency.

By the time most LGSOCs are diagnosed, they represent late-stage tumors with invasive growth [14]. However, non-invasive precursor lesions, known as serous borderline tumors (SBTs), account for 10–15% of ovarian epithelial malignancies [15]. They generally have an excellent prognosis, with OS only slightly lower than that of the general population [15]. In general, SBTs are clinically different from LGSOCs [16], but they share the same *KRAS* and *BRAF* mutations [15], consistent with evidence defining them as precursors of clinically defined LGSOCs [17]. Whereas most LGSOCs are diagnosed at stage III [14], the majority of SBTs are detected at stage I [18]. Given the nearly identical gene expression profiles between SBTs and LGSOCs [19], the continuum of disease from SBT to LGSOC [8, 20], the late-stage detection of LGSOCs [21], and noninvasive phenotype of SBTs, we chose to compare SBTs rather than LGSOCs to HGSOCs.

Our hypothesis was that regulatory differences between HGSOCs and SBTs would highlight factors and molecular processes involved in the invasive growth of HGSOCs. We also hypothesized that differential gene expression, utilized as a first step in discovery, would identify sets of co-regulated genes—and by virtue of these genes’ co-regulation, shared transcription factors whose role in malignant tumor growth could be further evaluated. Therefore, in a discovery set of 4 SBTs, 3 stage II HGSOCs, and 4 stage III HGSOCs, we identified differentially expressed genes (DEGs). We then performed expression-level validation testing in an independent dataset, enrichment analysis of binding motifs, and ChIP-seq analysis in an ovarian cancer cell line, PEO4. Our findings are the first to implicate the MegaTrans complex, originally discovered in breast cancer, in ovarian cancer as well. The Megatrans complex regulates estrogen-responsive molecular networks in a complex fashion [22, 23]. We conclude that the multidimensional nature of this potent complex could play a role in the heterogeneity of ovarian cancer.

## Materials and methods

### Sample collection and preparation

We obtained ovarian tumor RNA from the Magee-Womens Hospital Tissue Procurement Program (Pittsburgh, PA), where all samples were de-identified prior to receipt. Samples included 4 SBTs and 12 HGSOCs (stages I-III). The tissues were snap-frozen after surgery at the Magee-Womens Hospital and stored at − 80 °C. Total RNA was isolated using the QIAGEN RNeasy kit (Germantown, MD). Total RNA integrity (determined by RNA Integrity Number; average 8.0) was checked using an Agilent Bioanalyzer (Santa Clara, CA), and RNA purity (assessed by 260/280 ratio; average 1.8) was determined using a Thermo-Fisher Scientific NanoDrop (Waltham, MA). DNA was isolated for 4 SBT samples and 12 HGSOC samples using the Qiagen QIAamp Mini kit (Qiagen, Carlsbad CA) following the manufacturer’s instructions. DNA quality was assessed using a SmartSpec Plus spectrophotometer (BioRad, Hercules, CA).

### Characterization of SBT mutations via whole exome and RNA-seq analyses

Four SBT samples were examined for sequence variants by comparing whole exome or transcriptome sequencing data to the human reference genome. To obtain whole exome sequences, whole exome libraries with ~ 280 base inserts and paired-end index adapters were prepared from 1 μg genomic DNA, according to Illumina’s TruSeq DNA Sample Preparation v2 method. Five hundred nanograms of each of four libraries were pooled together for enrichment. Exome capture was performed according to Illumina’s TruSeq Exome Enrichment Kit protocol. Each captured exome pool was sequenced in two lanes on a HiSeq 2000 using version 3 chemistry. At least 40 million paired-end 100 base reads were obtained for each sample. Data was processed using the Illumina data analysis pipeline RTA v1.13.48 and CASAVA v1.8.2.

### SBT and HGSOC DNA methylation analysis

Human methylation data were derived from Illumina 27K and 450K methylation array datasets and included 4 normal fallopian tube and 5 normal ovarian surface epithelial tissue samples (GSE81224), 8 normal fallopian tube samples from The Cancer Genome Atlas (TCGA) OVCA methylation dataset, and 12 HGSOCs and 4 SBTs from the Magee-Womens Hospital Tissue Procurement Program. For the tumor samples, bisulfite conversion was performed on 0.50 μg genomic DNA, as has been described previously [24]. The top 500 most variable positions, ranked by beta-value range, were examined in a hierarchical clustering analysis of SBTs, HGSOCs, and controls.

### Transcriptome sequencing

To obtain RNA-seq gene expression data, we used the Illumina Genome Analyzer IIx (GAIIx) platform (San Diego, CA, USA) to sequence the poly-adenylated fraction (mRNA) of 11 tumor samples (4 SBTs, 3 stage II HGSOCs, and 4 stage III HGSOCs). Using a Covaris, Inc. E210 ultrasonicator (Woburn, MA, USA), we fragmented sample mRNA before using it as a template for first-strand cDNA synthesis using random primers and Life Technologies, Inc. SuperScript II Reverse-Transcriptase (Carlsbad, CA, USA). We ligated Illumina adaptors to the ends of double-stranded cDNA fragments and then used the Sage Science Pippin Prep system (Beverly, MA) to select 500-bp final products. After size selection, library enrichment consisted of 10 to 12 PCR cycles. Using version 5 chemistry, we sequenced each library on a single lane of an Illumina GAII_x_ flow cell and used the Illumina pipeline for image analysis and base-calling. These methods resulted in 40 to 69 million 51-bp paired-end reads (median: 65 million reads) per tissue sample.

### Normalization and quantification of differential gene expression

Our goal was to produce two sets of DEGs: SBTs vs. HGSOCs and stage II HGSOCs vs. stage III HGSOCs. To do so, we followed the TopHat and Cufflinks protocol [25] for RNA-seq analysis. RNA-seq reads were mapped against the human reference genome (build hg19) using TopHat v2.0.9 [26] and assembled into aligned read files using Cufflinks v2.1.1 [25]. We quantified gene expression values as fragments per kilobase of transcript per million mapped reads. To prevent bias (i.e., false negatives) resulting from missing data, we removed from the analysis genes in the Cufflinks outputs that were not present in all samples. We took the intersection of reported genes across all samples, resulting in a total of 13,208 genes. Using Cuffmerge, we combined outputs from Cufflinks into a master transcriptome to input into Cuffdiff [25]. We used Cuffdiff and Student’s t-tests to identify DEGs.

### Data visualization and clustering

To cluster gene expression associated with each sample subtype, we performed principal component analysis (PCA) using a covariance matrix and hierarchical cluster analysis, using the Euclidean distance metric in R statistical software. We generated all PCA and box plots using the ggplot2 package in R statistical software.

### Gene ontology

Ontology enrichment was determined using the 5 T (tree-travel, transform, t-test) method [27]. Briefly, first we extracted the gene ontology (GO) identifier from GO annotations for the expressed genes (fpkm > 0). Then we constructed an in-group gene list and an out-group gene list, where “in-group” entries were the genes within a given GO term and “out-group” entries were the remaining genes. For each GO term, we applied a t-test to the in-group and out-group expression values and repeated the process for all functional groups (as described in [27]). RNA-seq expression data for each sample type (SBT, stage II HGSOC, and stage III HGSOC) was used to identify the most notable terms defining each group.

### Gene fusion analysis

To detect recurrent gene fusion events in our samples, we analyzed transcriptome sequence data with EricScript [28] using its default parameters. The EricScore, or the probability of genuine fusion junctions, ranges from 0 to 1. The EricScore is a composite of three scores: genuine junction score, edge score, and uniformity score, which provide confidence for genuine fusion products. EricScript produces a single score using an AdaBoost classifier, which enabled us to rank predicted gene fusions by score strength and produce a list of fusions scoring higher than 0.50.

### Machine learning assessment of classification accuracy in a validation dataset

We performed an in silico evaluation of the DEGs’ ability to reproducibly classify tumor-pathological subtype differences (such as those defined by the International Federation of Gynecology and Obstetrics, or FIGO) when used on validation datasets. Two machine learning techniques were used to assess the efficacy of classification: decision tree (DT) and random forest (RF). To address advantages and disadvantages of each machine learning approach, we applied three-fold nested cross-validation using both the DT and RF algorithms on the DEGs with scikit-learn 0.17 [29]. The outer loop was used to calculate accuracy and the inner loop was used to perform a grid-search to optimize the *min_samples_split* and *max_depth* parameters.

### Validation of proposed tumor-classification DEGs using published microarray data

To validate the ability of our identified DEGs to classify tumors, we tested them on an independent ovarian cancer gene expression dataset (GSE9891 [30]; downloaded from the Gene Expression Omnibus website). Dataset GSE9891 came from a study that identified six molecular subtypes of ovarian tumors and included gene expression levels for 18 SBTs and 267 HGSOC tumors generated from microarray analysis. We note that the probe intensities in GSE9891 were normalized and log2-transformed values [30]. For each gene in the dataset, we performed a Student’s t-test using the SciPy function *scipy.stats.ttest_ind* to assess the significance of expression differences based on the log-transformed values. For boxplot visualization, raw probe intensities were used.

### Validation of proposed tumor classification DEGs using RNA-seq data from TCGA

We also used TCGA data (OVCA) to assess classification of stage II (*n* = 13), stage III (*n* = 179), and stage IV (*n* = 32) HGSOC samples using the 17-gene signature identified earlier in the study. RSEM-normalized gene expression data from RNAseqV2 was downloaded from firebrowse.org with cohort = OV. HGSOC stage info was extracted from the file OV.clin.merged.txt. The aforementioned RF algorithm with nested cross-validation was used to evaluate the classification power of the 17-gene signature to discriminate stage II from stage III or IV samples. Gene expression profiles were also compared individually between stage II, III or IV samples using the Mann-Whitney U test.

### Overall and disease-free survival analysis

To assess the role of the differentially expressed and fusion genes in patient survival, we tested whether there was a statistically significant relationship of each gene to overall or disease-free patient survival using data from the TCGA OVCA cohort, obtained from the cBioPortal cancer data repository [31, 32]. Using the cBioPortal toolkit, we produced Kaplan-Meier survival curves for the candidate genes, setting thresholds for significance as z-scores with +/− 2.0 difference from the median expression value seen for the same gene in diploid tumors. *P*-values reported by the tool are from a logrank test.

### Promoter motif enrichment analysis

To investigate upstream regulators of the DEGs, we investigated motif enrichment in their promoter sequences. We defined promoters as the sequences located 2000 bp upstream of the transcription start sites (TSSs). We retrieved these sequences from Ensembl Biomart [33]. Promoters of two genes, *CRABP2* and *MAFB*, were not found in Ensembl Biomart and were thus excluded from the analysis. We then used an ensemble of five tools, Emotif-Alpha [34] (GimmeMotifs [35], DECOD [36], DME [37], gkm-SVM [38], and info-gibbs [39]) for motif discovery. The motif discovery inputs consisted of the 2-kb gene promoters of our selected foreground genes vs. promoters of a set of background genes. The background gene sets, four times the size of the foreground gene sets, were drawn from genes expressed in a human foreskin fibroblast cell line. This gene set was chosen because the genes are expressed independent of cytokine treatment [40], and cytokine expression can vary according to ovarian carcinoma stages [41]. The first motif discovery set contained the 17 DEGs from the analysis of stage II vs. stage III HGSOC samples as the foreground genes and 68 background genes selected randomly from the background gene set. The second motif discovery set contained the 9 DEGs (after excluding *CRABP2* and *MAFB*) from the analysis of SBT vs. HGSOC samples as the foreground genes and 36 genes selected randomly from the background set.

We used the Find Individual Motif Occurrences (FIMO) tool [42] to scan sequences for motifs. To find putative promoter elements, we established a motif-ranking method based on an RF classifier. The RF algorithm is an ensemble learning method; it uses bootstrap sampling techniques and constructs a DT for each sub-sample. Using the Python library scikit-learn [29], we ranked binding sites using Gini impurity [43] and information gain [44] criteria. We retained the union set of the top 20 motifs for each criterion and calculated foreground and background coverage for each motif. We filtered out motifs obtained from the first motif discovery set with less than 50% foreground coverage and more than 35% background coverage, leaving eight motifs. We filtered out the motifs obtained from the second motif discovery set with foreground coverage of 80% and background coverage of 35%, leaving seven motifs. Two of the seven motifs were similar; we removed the motif with lower foreground coverage from further analysis. We matched the de novo motifs to a known motif database, HOCOMOCO v10 [45], using TOMTOM [46] and applied the Euclidean distance similarity function (requiring *P*-value threshold < 0.001). Accuracy was calculated as the percentage of correctly identified promoters containing the motif occurrence out of all promoter sequences.

### Transcription factor binding analysis using ChIP-seq data from UniBind

To investigate transcription factor (TF) binding sites within the DEG promoter sequences, we examined the UniBind repository [47] and downloaded ChIP-seq data for ERα, FOXA1/F1, RARA, and BHLHE40/41 binding sites for each promoter region. Additionally, all available ChIP-seq factors within these promoter sequences, in any cell line, were collated and ranked by the frequency of occurrence in each dataset.

### Cell growth and cross-linking for ChIP-seq

To confirm predicted binding sites of the regulators FOXA1, PITX1, and ERα, we used PEO4 cells, derived from a poorly differentiated serous ovarian adenocarcinoma. PEO4 cells were obtained from Sigma-Aldrich (St. Louis, MO). Cells were grown to confluency in T75 flasks and crosslinked with 1/10th the volume of formaldehyde solution (Imquest Bio, Fredrick, MD). Flasks were agitated for 15 min at room temperature to fix the cells. Fixation was stopped by adding 1/20th volume of glycine solution to the existing media in the flask. The cultures sat at room temperature for 5 min. Following the incubation, cells were scraped from the flask, combined, and enumerated using trypan blue exclusion methodology. Two million cells were distributed in two conical tubes and pelleted by centrifugation at 800 x g at 4 °C for 10 min. The supernatant was removed, and the cells were resuspended in 10 mL chilled PBS-Igepal. The cells were pelleted a second time. The supernatant was removed, and the pellet resuspended in 10 mL PBS-Igepal. Next, 100 mL 1 mM phenylmethanesulfonyl fluoride was used to resuspend the pellet. The cells were centrifuged a third time, and the supernatant was removed entirely from the cell pellet. The cell pellets were snap-frozen on dry ice and stored at − 80 °C.

### ChIP-seq and analysis in the ovarian cancer cell line PEO4

To determine TF binding sites in ovarian cancer cells, we performed ChIP experiments on PEO4 cell chromatin, via services at Active Motif (Carlsbad, CA), utilizing their standard protocol. Antibodies for the TFs FOXA1 (Abcam, cat # ab5089), ERα (Millipore, cat number 06–935), and PITX1 (Bethyl, cat# A300-577A, Lot# A300-577A-2) were used. Each ChIP reaction was carried out using 30 μg PEO4 cell chromatin and anti-TF antibody. ChIP DNA was processed into a standard Illumina ChIP-seq library and sequenced to generate > 5 million reads. Reads were aligned to the human genome (hg38), and after removal of duplicate and non-uniquely mapped reads, ~ 25 million alignments were obtained. A signal map capturing fragment densities along the genome was generated and visualized in the Integrated Genome Browser. In addition, MACS peak finding was performed to identify the most significant peaks. Using a default cutoff of *P* = 1x10^-7^ (without control file), peaks were identified after ENCODE blacklist filtering. Between 1000 and 2000 peaks were identified for ERα replicates and 12,000–22,000 for PITX1 replicates. The average fraction of reads in peaks was 0.4% for ERα and 4.6% for PITX1.

## Results

### Molecular characterization of SBTs

The four SBTs available for transcriptome analysis in this study were subtyped prior to 2010, when they were classified as low malignant potential tumors according to the terminology then current. Because our ability to molecularly characterize EOC subtypes has improved since that time, we analyzed gene variants and methylation in these samples to verify that the sample characteristics were consistent with what is now known about SBTs. Whole exome sequencing and RNA-seq data showed that three of the four tumors harbored the BRAF V600E mutation, consistent with the mutation profile of SBTs [15].

**Fig. 1:**
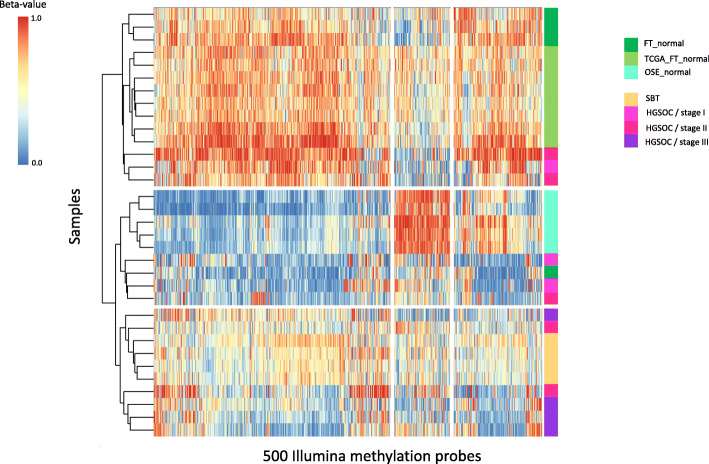
Hierarchical clustering of ovarian tumor and normal samples using methylation data shows that serous borderline tumors (SBTs) cluster with one another and are distinct from normal tissue samples. Clustering was performed on beta-values derived from Illumina 450 K methylation sites, selected by methylation beta-value range. Our study generated the methylation data for 4 SBTs and 12 high-grade serous ovarian cancers (HGSOCs), as well as 4 normal fallopian tube samples (FT_normal samples). Data from the published literature included 5 normal ovarian surface epithelium (OSE_normal) and 8 normal fallopian tube (TCGA_FT_normal) samples

In addition, we generated methylation microarray data and, using the beta-value range, determined differentially methylated CpG sites, as in Kolbe et al. [24]. The comparison included 4 SBTs, 12 normal fallopian tube (FT) samples (including 8 from TCGA), and 12 HGSOCs (stages I-III), with data from an additional 5 normal ovarian surface epithelial (OSE) cell samples added from the literature (GSE81224 [48]). When these sites were subjected to hierarchical clustering analysis, normal samples were divided into two major clades (Fig. 1): (*i*) normal FT samples and (*ii*) normal OSE cell samples. This latter clade had two prominent nodes, the first included OSE samples, a few stage I and II HGSOC samples, and one FT sample; the second included all four SBT samples, as well as stage II and all stage III HGSOC samples. HGSOC samples were found across all clusters, independent of their stage, suggesting intrinsic heterogeneity. These data indicate that the SBT samples were most similar to one another, consistent with the pathologist’s original classification, and that the SBT samples shared characteristics at these epigenetic sites with stage II and III HGSOCs.

### DEGs associated with invasiveness in EOC

#### Genes differentially expressed between SBTs and HGSOCs

**Fig. 2:**
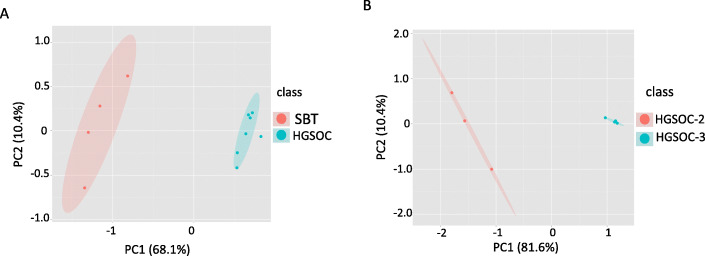
Principal component analysis (PCA) shows that differential gene expression distinguishes serous borderline tumors (SBTs) from high-grade serous ovarian cancers (HGSOCs) and stage II from stage III HGSOC samples. **a** PCA of expression at 11 genes differentially expressed between SBTs (*n* = 4) and HGSOCs (*n* = 7). Principal components 1 and 2 (PC1,2) are labeled with the percent variability they explain in the data. The areas enclosed by the ellipses represent the “within 80% confidence interval” under a bivariate t-distribution. **b** PCA of expression at 17 genes differentially expressed between stage II (HGSOC-2, *n* = 3) and stage III (HGSOC-3, *n* = 4) HGSOC samples

Using transcriptome data from 4 SBTs and 7 HGSOCs (3 stage II and 4 stage III samples) (Table S1), we investigated which genes were differentially expressed between the two EOC subgroups (Table 1). Eleven DEGs were identified, on the basis of a Student’s t-test (*P* < 0.05): 2 genes were upregulated in HGSOC and 9 were downregulated. PCA analysis demonstrated that expression levels of these 11 genes were sufficient to distinguish between SBTs and HGSOCs (Fig. 2a). Hierarchical clustering gave a similar result (Figure S1).

**Table 1:** Eleven genes differentially expressed between serous borderline tumors (SBTs) vs. combined stage II and stage III high-grade serous ovarian cancers (HGSOCs)

		SBT expression		HGSOC expression		^a^SBT/ HGSOC	
Gene name	Coordinates	Mean	Std dev	Mean	Std dev	Fold change	***P***-value
*SLC7A2*	chr8:17354596–17428077	65.35	16.00	1.99	2.09	32.84	2.00x10^−6^
*PIFO*	chr1:111889194–111895639	55	35.4	1.9	1.44	28.95	2.53x10^− 3^
*AFF2*	chrX:147582138–148082193	3.2	2.24	0.24	0.53	13.33	7.12x10^−3^
*HES2*	chr1:6475293–6479979	5.1	2.44	0.59	0.49	8.64	8.24x10^−4^
*BBS12*	chr4:123653856–123666098	4.11	1.16	0.69	0.47	5.96	5.90x10^−5^
*RPL12*	chr9:130209952–130213711	1565.46	698.27	301.98	168.7	5.18	1.07x10^−3^
*RPL7A*	chr9:136215068–136218280	227.97	89.21	45.48	31.97	5.01	6.98x10^−4^
*RPS15*	chr19:1438362–1440492	3218.63	1563.63	708.2	547.09	4.54	3.22x10^−3^
*RPS12*	chr6:133135707–133138703	3056.61	1034.17	708.02	630.41	4.32	1.04x10^−3^
*MAFB*^b^	chr20:39314516–39317876	5.78	2.56	21.04	14.94	−3.64	7.89x10^−2^
*CRABP2*^b^	chr1:156669399–156675608	20.13	19.77	119.77	87.65	−5.95	5.59x10^−2^

^a^For any value smaller than 1 (i.e. for downregulation), the fold change value was replaced by its negative reciprocal value. ^b^These two genes exhibited 3- to 6-fold higher mean expression in HGSOC. They were included in the table because they have *P*-values close to the threshold and have been linked to cancer in previous studies

#### Genes differentially expressed between stage II and stage III HGSOCs

Next, we compared transcriptome expression data between the 3 stage II and 4 stage III HGSOCs. This time, 17 significant DEGs were identified, all downregulated in stage III HGSOCs (Table 2). PCA demonstrated that expression levels of the 17 genes were sufficient to distinguish between stage II and stage III HGSOCs (Fig. 2b). Hierarchical clustering gave a similar result (Figure S1).

**Table 2:** Seventeen genes differentially expressed between stage II vs. stage III high-grade serous ovarian cancer (HGSOC) samples

		Stage II expression		Stage III expression		II/III	
Gene name	Coordinates	Mean	Std dev	Mean	Std dev	Fold change	***P***-value
*TSPAN1*	chr1:46640748–46651634	94.34	22.3	5.38	5.46	17.55	5.20x10^−4^
*ANK1*	chr8:41510743–41754280	0.46	0.18	0.04	0.06	10.18	6.61x10^−3^
*SERPINE2*	chr2:224839764–224904036	68.45	45.04	7.46	6.57	9.18	3.98x10^−2^
*SYNPO*	chr5:149980641–150038792	46.57	20.07	5.24	4.47	8.89	9.24x10^−3^
*PDGFC*	chr4:157682762–157892546	30	18.76	3.77	0.85	7.95	3.42x10^−2^
*NQO1*	chr16:69743303–69760533	20.81	9.36	2.79	1.59	7.45	1.14x10^−2^
*CA12*	chr15:63615729–63674075	30.59	8.42	4.41	3.49	6.94	2.25x10^−3^
*PPP1R14C*	chr6:150464187–150571528	4.32	1.04	0.63	0.23	6.86	8.60x10^−4^
*TMEM30B*	chr14:61744088–61748530	14.47	4.55	2.2	1	6.59	2.96x10^−3^
*PGK1*	chrX:77359665–77382324	147.96	70.15	25.3	10.29	5.85	1.62x10^−2^
*DNAJB9*	chr7:108210188–108215294	13.32	8.54	2.45	0.76	5.45	4.70x10^−2^
*CLIC1*	chr6:31698357–31704341	236.93	57.2	48.63	6.54	4.87	1.08x10^−3^
*ECE1*	chr1:21543739–21672034	25.1	4.53	5.67	3.03	4.42	1.00x10^−3^
*ENPP4*	chr6:46097700–46114436	3.17	0.88	0.74	0.35	4.26	3.71x10^−3^
*ZDHHC7*	chr16:85008066–85045141	26.42	14.78	6.32	2.57	4.18	4.02x10^−2^
*GCLC*	chr6:53362139–53409927	9.83	3.68	2.39	0.75	4.11	9.78x10^−3^
*CHST15*	chr10:125767181–125853123	13.95	0.82	3.57	1.39	3.9	9.00x10^−5^

#### Biological properties of DEGs

Next, we used gene ontology to analyze the biological properties specific for each tumor subtype and stage (Table S2; see Materials and methods), as well as the properties of the DEGs identified in the two comparisons described above. SBT gene expression was enriched for inflammatory pathways, including chemokine production, regulation of leukocyte activity, dopachrome isomerase activity (associated with PD-L1 upregulation [49]), and zinc ion sequestration, corresponding to negative regulation of mature B-cell apoptotic processes. Stage II HGSOC gene expression was enriched in chromatin and transcriptional regulatory processes, including those that involve histone acetyltransferase and histone methyltransferase complexes. Stage III HGSOC gene expression was enriched in actin cytoskeleton, cell membrane, and plasma membrane organization. Seven of the 11 DEGs identified in the SBT vs. HGSOC comparison were involved in biological processes involving pre- and post-transcriptional regulatory processes (*AFF2*, *MAFB*, *HES2*, *RPL12*, *RPL7A*, *RPS12*, *RPS15*), whereas 5 of the 17 DEGs identified in the stage II vs. stage III HGSOC comparison were involved in the biological processes of cell migration and cytoskeletal organization (*PDGFC*, *SERPINE2*, *TSPAN1*, *ANK1*, *SYNPO*). These data, in which cell migration emerges as a theme in the stage III HGSOC gene ontology analysis, invoke a molecular explanation for the separation of the tumor subtypes and stages that explains the known correlation between increasing stage and invasiveness.

#### STAG3 gene fusions

In addition to identifying DEGs, we investigated whether gene fusions play an important role in invasiveness by comparing the SBT and HGSOC samples. Using the transcriptome sequence data, we identified 5 fusions occurring in all SBTs, 15 in all stage II HGSOCs, and 12 in all stage III HGSOCs (see Tables S3, S4 and S5 for all fusion genes); singleton fusion occurrences were recorded but not counted here. Strikingly, all samples demonstrated a fusion event in *STAG3.*

#### Classification accuracy of DEGs in microarray data from an SBT and HGSOC validation sample

**Fig. 3:**
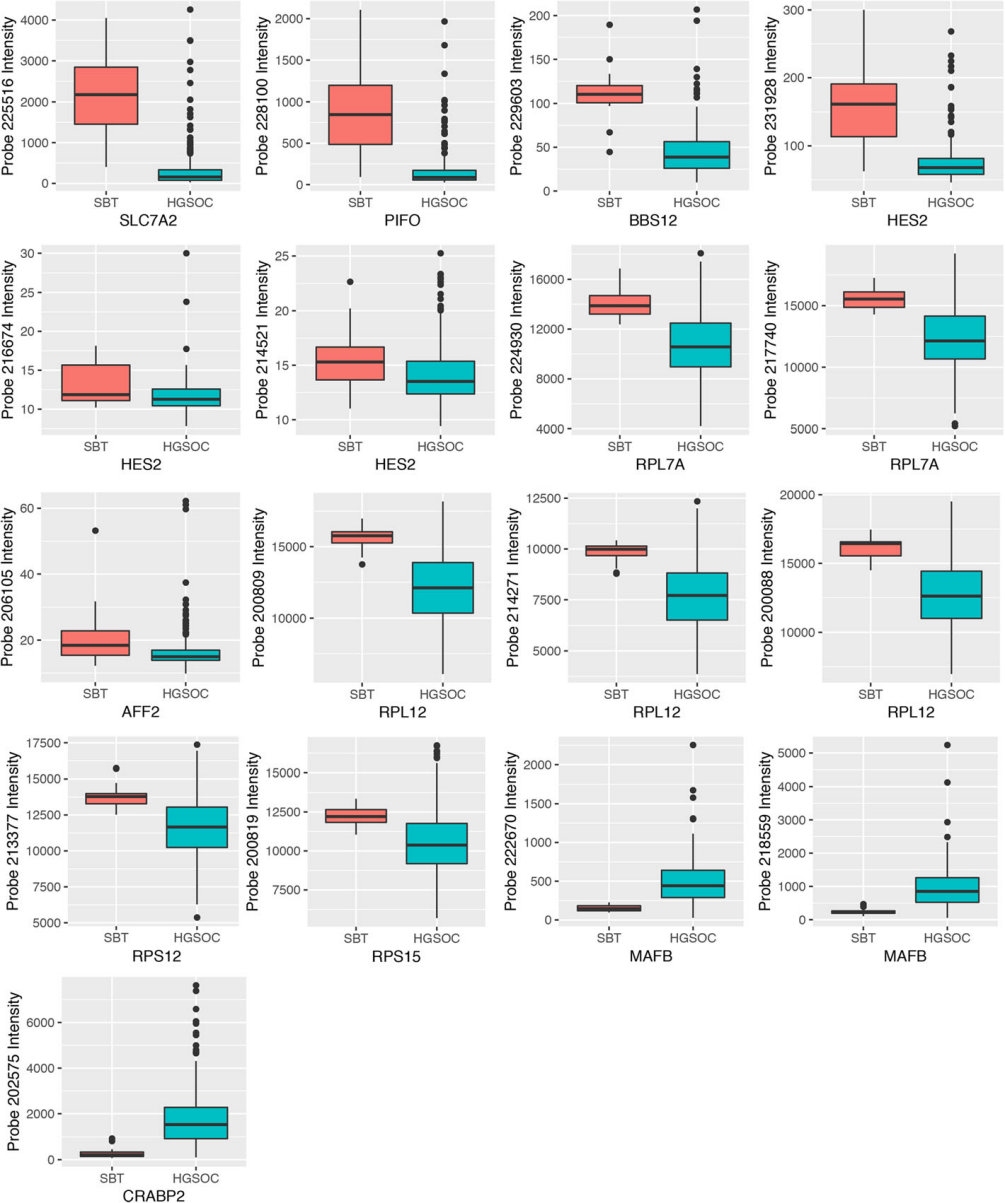
Validation testing of 11-gene signature for distinguishing between serous borderline tumors (SBT, red boxes) and high-grade serous ovarian cancers (HGSOC, blue boxes). Expression level differences between SBTs and HGSOCs at the 11 genes remained significant (*P* < 0.05) in the validation dataset GSE9891, which consists of microarray expression data from 18 SBT and 267 HGSOC samples. Labels on the y-axis show the probe names, and the values represent probe intensity. Individual genes may be represented by more than one probe on the array, in which case multiple plots are shown per gene

To determine whether expression levels at these DEGs could reliably be used to classify the two EOC subtypes, we tested them in a validation set consisting of microarray data for 18 SBT and 267 HGSOC samples (dataset GSE9891). To minimize overfitting the data, we employed two different machine learning methods, decision tree (DT) and random forest (RF), and compared their output. The 11-gene signature for distinguishing between SBT and HGSOC samples yielded classification accuracies of 95.1% with DT and 97.9% with RF. We also asked if each of the 11 individual genes was significantly differentially expressed and if the directionality of fold enrichment remained unchanged. Expression differences at 11 genes met the significance threshold (*P* < 0.05), with the same directionality seen previously (Fig. 3; Table S6): 9 were downregulated in HGSOC and 2 genes were upregulated. Additionally, 7 genes showed absolute fold changes ≥ 2 between SBT and HGSOC samples.

#### Further validation of DEGs for use in staging HGSOC tumors

Next, we used the RF approach to test the ability of the 17 DEGs identified in the stage II vs. stage III HGSOC comparison to discriminate between stage II, III, and IV HGSOCs, using 563 samples from the TCGA OVCA RNA-seq dataset. The ability of the 17-gene signature to correctly classify stage II vs. stage III samples was not significant (*P* > 0.05); however, we were able to validate a significant change in expression in one of the 17 DEGs in the TCGA data. *GCLC* (glutamate-cysteine ligase synthetase catalytic subunit) displayed lower expression in stage IV samples than in stage II samples (*P* = 8.98x10^-3^, Mann Whitney U test) (Figure S2), but expression was not significantly different in stage III vs. stage II TCGA data. The directionality of the difference in *GCLC* between stage IV vs. stage II samples was the same as in the discovery dataset, where a decrease was also seen in stage III (2.39) compared to stage II (9.83) tumors, as shown in Table 2. In sum, validation failed for stage II vs. stage III classification using the TCGA OVCA dataset, in contrast to our success at validating SBT vs. HGSOC classification using the GSE9891 dataset. This failure may be due to extensive similarity in HGSOC stage II and III. It could also be due to nuanced differences in staging criteria used for EOCs in TCGA samples vs. our samples (which were collected prior to 2010), as explained in Duska et al. [50]. It could also be due to heterogeneity of the tumor collections, demonstrating the need to develop more sensitive biomarkers for the molecular staging of ovarian tumors. Of note, *GCLC* is a promising therapeutic target in cancer [51].

#### Association between DEGs and survival in patients with HGSOC

**Fig. 4:**
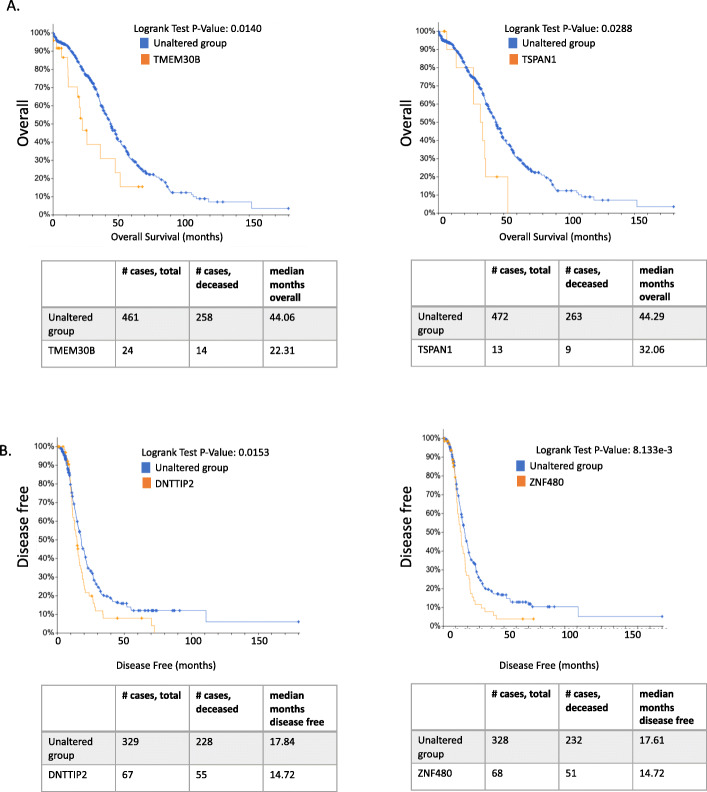
Association between survival and expression levels of differentially expressed and fusion genes identified in a comparison of serous borderline tumor (SBTs) and high-grade serous ovarian cancers (HGSOCs), among 563 patients with HGSOC. Shown are 4 genes associated with either **a** differential expression or **b** gene fusions in our discovery dataset, whose altered expression was significantly associated with survival. Expression and survival data were obtained from the Cancer Genome Atlas (TCGA) ovarian carcinoma dataset [31]. The y-axis labels indicate whether overall survival or disease-free survival is shown. Here, altered expression is defined as being ≥2 standard deviations from the mean expression level of all diploid samples in the TCGA ovarian cancer collection. Additional genes are included in Figure S3

To determine whether any of the 28 DEGs or genes associated with gene fusion events might have clinical significance, we examined the association between their expression and disease-free survival (DFS) or OS in a larger cohort of 563 TCGA HGSOC samples (from the TCGA OVCA dataset), using cBioPortal [32]. The expression levels of two genes identified from our DEG analysis, *TMEM30B* and *TSPAN1*, were significantly associated with OS (*P* = .014–0.028; Fig. 4a). The expression levels of three genes associated with gene fusion events (*DNTTIP2*, *ZNF480*, and *BPTF*) were associated with DFS (*P* = .008–0.016; Fig. 4b and Figure S3). The expression levels of three other genes associated with gene fusion events (*SPINT2, FCRL5*, and *STAG3*) were associated with OS (*P* = .008–0.021; Figure S3). For comparative purposes, we also assessed the association between *TP53* mutations—the most frequently occurring mutational events in HGSOC, reported in ≥ 96% of samples [31]—and DFS and OS. Given the prevalence of *TP53* mutations in HGSOC samples, one might expect it to be a good predictor of survival; however, unlike the alterations identified above, *TP53* mutations (at all positions) were not significant predictors of either DFS or OS.

### Prediction of transcription factor (TF) binding sites in DEG promoters

To determine whether expression of any of the 28 DEGs identified in our study might be governed by shared regulatory pathways, we investigated shared regulatory motifs across the associated promoters using an ensemble-based motif discovery method, Emotif-Alpha [34]. Each foreground motif was compared to a four-fold larger background set of sequences (drawn from genes expressed in a human foreskin fibroblast cell line). The results indicated significant TF enrichment in each dataset: 6 candidate TF binding site motifs were matched to known factors in the SBT vs. HGSOC comparison (Table S7) and 8 candidate TF binding site motifs were matched to known factors in the stage II vs. stage III HGSOC comparison (Table S8). The enrichment of these TF motifs suggests they may have a role in co-regulating the affected genes.

**Fig. 5:**
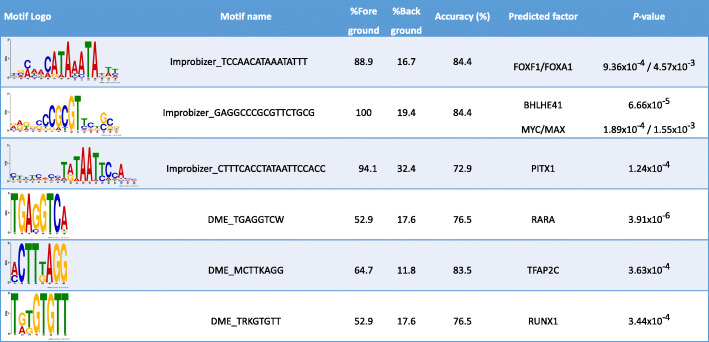
Transcription factors (TFs) implicated in the differential expression of genes associated with invasiveness in epithelial ovarian cancer. We identified TF motif enrichment in the promoter sequences of the differentially expressed genes, relative to background sequences. Motif names were determined by comparing motifs to known TF sequence logos (see Materials and methods). TFs matching the same logos are presented in the same row, with the same percentage values. *P*-values represent the significant match to the known motif. The matches to MYC/MAX, TFAP2C, and RUNX1 were discovered secondarily, from their high frequency of occurrences in the UniBind analysis portion of this study. CRABP2 and MAFB promoters were excluded due to lack of sequence annotations in Ensembl Biomart

To identify the TFs capable of binding the enriched motifs that we detected, we conducted a similarity search to a database of known human TF sequence logos. We found significant matches for FOXF1, BHLHE41, PITX1, and RARA (Fig. 5). Two additional related TFs were also implicated: FOXF1 has a family member, FOXA1, which could recognize the same motif, with slightly reduced predicted binding specificity (*P* = 9.36x10^-4^ for FOXF1; *P* = 4.57x10^-3^ for FOXA1). In addition, BHLHE41 has a paralog and binding partner, BHLHE40, which is an additional candidate for binding at the same motif. Individual motif occurrences localized BHLHE41/BHLHE40 binding to positions 1–2 kb upstream of the transcription start sites (TSSs), whereas FOXF1/FOXA1 was localized to positions < 1 kb from the TSSs (Figure S4).

### Validation of predicted TF binding sites in DEG promoters

#### ChIP-seq analysis of cell lines represented in UniBind repository for predicted TFs

To determine whether any of the TFs identified in our analysis could occupy the genomic locations associated with the predicted binding motifs, we examined ChIP-seq binding data for FOXF1/FOXA1, BHLHE41/BHLHE40, PITX1, and RARA from the UniBind repository. This repository contains information for numerous cell types [47] but has little data from ovarian cancer cell lines. Although binding data for FOXF1, BHLHE41, and PITX1 was not available, we confirmed the binding of BHLHE40 at 10 DEG promoters, FOXA1 at 12 DEG promoters, and RARA at 3 DEG promoters (Table S9) when we surveyed data from all cell types.

#### ChIP-seq analysis of cell lines represented in UniBind repository identifies additional TFs

Using additional ChIP-seq datasets in the UniBind repository, we examined other factors binding within the DEG promoters. We found evidence of CTCF binding at 19 of the 28 promoters; MYC and MAX binding at 18 and 19 promoters, respectively; TFAP2C/AP2γ binding at 16 promoters; and RUNX1 binding at 11 promoters; as well as evidence of binding by numerous additional TFs at lower frequencies (Table S9). We confirmed that the binding motifs of MYC/MAX (same as BHLHE41), RUNX1, and TFAP2C/AP2γ matched sequence logos identified in our motif enrichment study (Fig. 5).

#### ChIP-seq analysis of cell lines represented in UniBind repository for ERα

A literature search of the biological roles of PITX1, FOXA1, and RARA indicated that each factor could interact with estrogen receptor α (ERα) [52–55]. Therefore, we searched the UniBind ChIP-seq data for evidence of ERα binding in the promoter regions of the 28 DEGs. We found evidence of ERα binding in 13 of the DEG promoters (Table 3, Table S9), although the ChIP-seq data was collected from breast cancer (MCF7) and endometrial cancer (Ishikawa) cell lines.

**Table 3:** Proof of concept, ERα binding in the promoter regions of 28 differentially expressed genes (DEGs)

DEG comparison	Proportion of DEGs with ERα binding	DEGs with ERα binding^a^
SBT vs HGSOC	4/11	*HES2, RPL12, RPS15, SLC7A2*
Stage II vs stage III HGSOC	9/17	*ANK1, CA12, CHST15, ECE1, NQO1, PDGFC, PPP1R14C, SERPINE2, TSPAN1*

*Abbreviations*: *HGSOC* High-grade serous ovarian cancer, *SBT* Serous borderline tumor

^a^ ERα binding determined using ChIP-seq data obtained from the UniBind repository from a breast cancer cell line (MCF7) and an endometrial cancer cell line (Ishikawa cells)

#### ChIP-seq data for FOXA1, PITX1, and ERα in ovarian cancer cell line PEO4

**Fig. 6:**
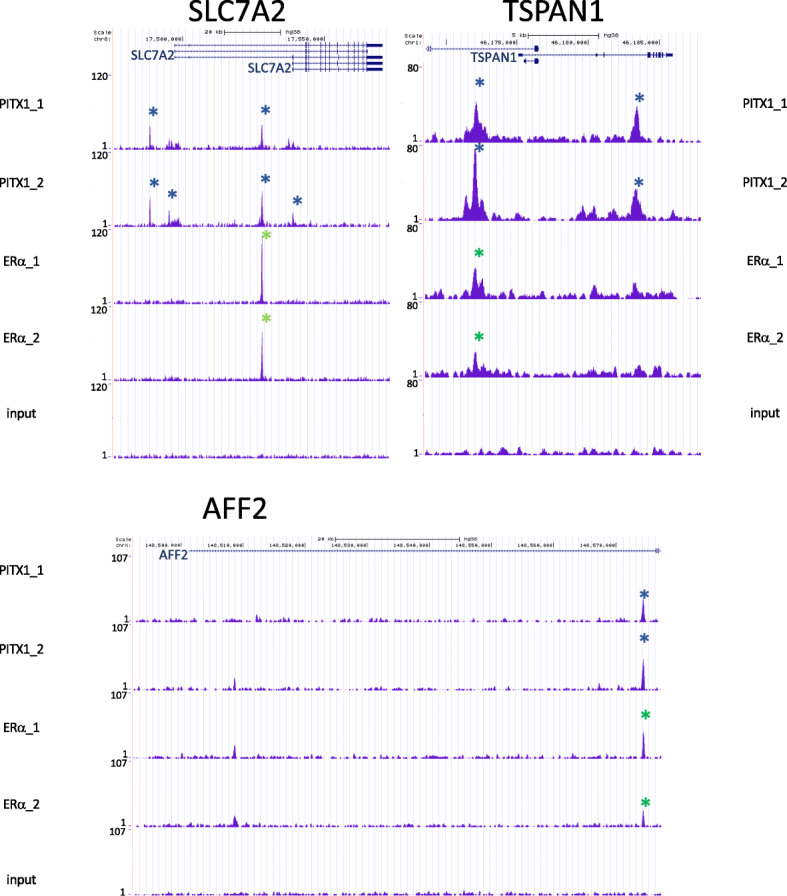
Experimental ChIP-seq data from the ovarian cancer cell line PEO4 illustrates transcription factors PITX1 and ERα co-occupying the promoter and gene bodies of three genes differentially expressed between epithelial ovarian cancer subtypes and stages. *SLC7A2* and *AFF2* were differentially expressed between serous borderline tumors (SBTs) and high-grade serous ovarian cancers (HGSOCs); *TSPAN1* was differentially expressed between stage II and stage III HGSOCs. Binding data are displayed on tracks from the UCSC Human Genome Browser. Two experimental replicates were performed for each transcription factor, along with an input control. Blue asterisks indicate PITX1 binding and green asterisks ERα binding, where peaks displayed statistical significance above background for the relevant transcription factor. The vertical viewing axis was assigned the value of the highest peak and kept consistent for all panels at each locus

To confirm the binding of our identified TFs in ovarian cancer, we performed ChIP-seq experiments for FOXA1*,* PITX1*,* and ERα in the ovarian cancer cell line PEO4, derived from a poorly differentiated serous adenocarcinoma [56]. We included ERα and FOXA1 (in lieu of FOXF1) based on their known relevance and protein-protein interactions in breast cancer, and PITX1 for relevance in other cancers and its novelty in ovarian cancer. Significant PITX1 binding occurred in 16 of the 28 DEGs, in at least one of two experimental replicates: 5 of 11 DEGs from the SBT vs. HGSOC comparison (Table 4) and 11 of the 17 DEGs in the stage II vs. stage III HGSOC comparison (Table 5). We show examples of significant ERα and PITX1 binding in *SLC7A2*, *TSPAN1*, and *AFF2* from both ChIP-seq replicates (Fig. 6). Collectively, these data indicate that the TF binding predicted from the DEG analysis can occur in ovarian cancers with similarity to PEO4 cells. In contrast to the results for PITX1 and ERα, repeated assessment of FOXA1 using two different antibodies failed to confirm binding of the protein. We note that DEGs from both the SBT vs. HGSOC and stage II vs. stage III HGSOC comparisons show binding of PITX1, which was not predicted in our motif logo enrichment testing, possibly due to small sample sizes.

**Table 4:** PITX1 and ERα ChIP-seq binding in the ovarian cancer cell line PEO4, in genes differentially expressed between serous borderline tumors vs. high-grade serous ovarian cancers

Number	Gene	PITX1 replicate 1	PITX1 replicate 2	ERα replicate 1	ERα replicate 2
1	*SLC7A2* ^a^	YES	YES	YES	YES
2	*BBS12*	–	–	–	–
3	*RPL7A*	–	–	–	–
4	*HES2*	–	–	–	–
5	*RPS12*	–	YES	YES	–
6	*RPL12*	–	–	–	–
7	*PIFO*	–	–	–	–
8	*RPS15*	–	YES	–	–
9	*AFF2*	YES	YES	YES	YES
10	*CRABP2*	YES	YES	YES	–
11	*MAFB*	–	–	–	–

^a^ ERα binding at *SLC7A2* was also present in UniBind data, though UniBind data was only available from breast and uterine cell lines. See Table 3

**Table 5:** PITX1 and ERα ChIP-seq binding in the ovarian cancer cell line PEO4, in genes differentially expressed between stage II and stage III high-grade serous ovarian cancer samples

Number	Gene	PITX1 replicate 1	PITX1 replicate 2	ERα replicate 1	ERα replicate 2
1	*CHST15*	–	–	–	–
2	*TSPAN1* ^a^	YES	YES	YES	YES
3	*PPP1R14C*	YES	YES	–	–
4	*ECE1*	YES	YES	–	–
5	*CLIC1*	–	–	–	–
6	*CA12* ^a^	YES	YES	YES	–
7	*TMEM30B*	–	YES	–	–
8	*ENPP4*	–	–	–	–
9	*ANK1*	–	YES	–	–
10	*SYNPO*	–	–	–	–
11	*GCLC*	–	YES	–	–
12	*NQO1*	YES	YES	–	–
13	*PGK1*	–	–	–	–
14	*PDGFC*	YES	YES	–	–
15	*SERPINE2*	–	YES	–	–
16	*ZDHHC7*	–	–	–	–
17	*DNAJB9*	–	YES	–	–

^a^ ERα binding at *SLC7A2* was also present in UniBind data, though UniBind data was only available from breast and uterine cell lines. See Table 3

#### Summary of motif enrichment and ChIP-seq data from UniBind and experimental analysis

Our motif enrichment analysis of the DEG promoters identified in the SBT vs. HGSOC and stage II vs. stage III HGSOC comparisons implicated 4 known TFs (PITX1, BHLHE40/41, FOXF1/A1, and RARA), as well as 10 unknown TFs. Our ChIP-seq data from the ovarian cancer cell line PEO4 showed that 16 of the 28 DEGs had statistically significant evidence of PITX1 binding. Furthermore, we integrated ERα ChIP-seq data from two sources, the UniBind repository and our experimental analysis in the PEO4 cell line. Using the combined data, we found 16 of 28 DEGs had statistically significant evidence of ERα binding—always in the presence of PITX1 in the PEO4 cell line (Tables 3, 4 and 5). This binding appeared in both SBT vs. HGSOC and HGSOC stage II vs. stage III DEGs; however, only three promoters, those of *TSPAN1*, *SLC7A2*, and *CA12*, overlapped between the ERα ChIP-seq data from our experiment in the PEO4 cell line and UniBind, indicating that context matters. Additional TFs capable of binding these promoters—CTCF, MYC/MAX, RUNX1, and TFAP2C—were found by comprehensively analyzing the UniBind ChIP-seq data for the 28 DEG promoters (Table S9). UniBind contains numerous, non-ovarian cancer cell types. Thus, the TF binding does not appear to be exclusive to a tumor-stage or subtype, but dynamically utilized among the DEGs in different cell types. Collectively, the suite of TFs identified suggests the existence of a larger regulatory network that governs expression of the genes responsible for EOC invasiveness through regulated differential binding.

## Discussion

HGSOCs represent an invasive EOC phenotype treated with surgery and chemotherapy, while SBT is non-invasive, more indolent, and treated with surgical resection alone. Our objective in this study was to molecularly contrast these two EOC subtypes, with the goal of identifying biomarkers that could be used to distinguish between the divergent processes responsible for differences in growth patterns, which include aggression and invasion. We performed gene expression profiling and then used promoter sequences of the DEGs identified to discover enriched regulatory motifs. Most of the DEGs identified (20/28) had not previously been reported to be involved in invasive epithelial ovarian cancer. Nevertheless, they could be used to classify independent samples by subtype with high accuracy (95.1% with DT and 97.9% with RF), and expression levels of two of them, *TMEM30B* and *TSPAN1*, were also predictors of OS.

We also highlight a number of genes affected by fusion events. Some of these genes were significantly associated with OS using TCGA patient data and may therefore be biologically relevant; thus, we have included them for the completeness of our ovarian cancer study. Fusion genes are not typically examined as part of differential gene expression studies and therefore are often missed. We found evidence of PITX1 and ERα binding in only one fusion gene, *SPINT2*, indicating that the majority of the fusion genes identified in this study contribute to ovarian cancer by mechanisms other than this regulatory network. Further analysis is needed to address these genes’ specific roles in ovarian cancers.

Of the 28 DEGs identified in our study, several are known ovarian cancer-related genes. In the SBT vs. HGSOC comparison, such genes included *SLC7A*, *PIFO*, *RPL7A*, and *CRABP2* [57–61] (Table S10). In fact, *SLC7A2* serves as a potential biomarker and therapeutic target for ovarian cancer [62]. The remaining seven DEGs in the SBT vs. HGSOC comparison, while not previously reported in association with ovarian cancer, have cancer or disease-related functions. For example, *HES2, MAFB, RPS12, RPL12*, *RPS15,* and *AFF2* are all associated with cancer [63–66], whereas *BBS12* is a gene related to Bardet-Biedl Syndrome, a multi-organ genetic disease that can involve hypoplasia of the uterus, ovaries, and fallopian tubes [67]. These findings support the biological plausibility of the candidate genes that we identified.

Similarly, 4 of the 17 DEGs identified in the stage II vs. stage III HGSOC comparison are known ovarian cancer-related genes: *TSPAN1*, *CLIC1*, *NQO1*, and *DNAJB9* [68–72] (Table S10). Notably, *TSPAN1* shows pronounced expression in serous ovarian carcinomas at FIGO stage IIIC [68, 69]. Two of the known ovarian cancer-related genes are also associated with ovarian cancer prognostic outcomes (*CLIC1* and *NQO1*) [69, 70]. Finally, 12 of the DEGs have known associations with other cancer types [73–80]. Of note, *ZDHHC7*, not previously identified to play a role in ovarian cancer, encodes a zinc finger protein that regulates a tumor suppressor important for establishing and maintaining epithelial cell polarity [81]. Therefore, the lower *ZDHHC7* expression in stage III vs stage II samples is consistent with reduced tumor suppressor activity.

Using in silico and in vitro analyses of the DEG promoter sequences, we identified multiple TF binding motifs and validated PITX1 and ERα binding using an ovarian cancer cell line. Many of the TFs we identified, including PITX1, RARA, FOXA1, and BHLHE41/E40 (including overlapping MYC/MAX sites), are known to interact with ERα, and many belong to the previously described MegaTrans complex [22], which has been shown to play an important role in aberrant ERα-regulated gene expression in breast cancer [23]. Many of the TFs we identified as putative regulators of the DEGs have also already been reported to be involved in oncogenesis. Notably, nuclear expression of TFAP2C has been associated with ovarian tumor aggressiveness [82]. However, of the four TFs (PITX1, RARA, FOXF1/A1, and BHLHE41/40) that matched the binding motifs in DEG promoters, only one, FOXA1, has previously been implicated in ovarian cancer specifically [83]. All four TFs do have documented roles in cancer cell mobility or invasiveness [84–87]. In particular, proteins BHLHE40/41 function as ligand-dependent co-repressor retinoic acid (RXR/RAR) heterodimers [88], indicating a known biochemical association between proteins binding two of our predicted TF motifs (BHLHE40/41 and RARA).

Our findings also point toward a potentially important role for ERα in determining EOC invasiveness. ERα is expressed at high levels in roughly 80% of HGSOCs, though it is not sufficient to predict therapeutic response [89]. Its interactions with factors identified in this study led us to predict and confirm ERα binding at the DEGs. For example, RARA binds cooperatively with ERα to regulate transcription at target sites within chromatin in breast cancers [52]. In addition, FOXA1 plays a central role in almost all ERα-chromatin interactions and gene expression changes in hormone-sensitive and -resistant breast cancer cell lines [54]. We could not detect FOXA1 binding in the ovarian cancer cell line PEO4 using ChIP-seq; it may only be needed for short-term activity as a pioneer factor, or it could be inaccessible within a larger protein complex, or not present in the cells. Finally, *PITX1* is under primary transcriptional control of ERα in breast cancer cells, and the protein it encodes is recruited to ERα-bound enhancers to modulate the transcriptional activity of the associated genes [53]. *PITX1* has been reported in a breast cancer repressive complex that contains RARA, FOXA1, and ERα; identified prior to MegaTrans [54]. Previous findings, paired with our results, suggest an analogous regulatory complex in ovarian cancer.

We identified fusions of the gene *STAG3*, which encodes meiotic cohesion protein, cohesin subunit SA-3, in all samples. This protein regulates the cohesion of sister chromatids during cell division. To investigate the biological relevance of this gene in an independent dataset, we examined *STAG3* expression using RNA-seq expression data from the TCGA OVCA cohort and found aberrant upregulation in 33 of 563 HGSOC tumors. Here, aberrant expression was defined as being ≥2 standard deviations from the median expression level of all unaffected samples in the TCGA OVCA collection. Of note, STAG3 loss of function is associated with premature ovarian failure [90], and a common allele is associated with elevated risk of developing EOC [91]. Mutant STAG3 has also been implicated in metastatic melanoma [92, 93], where it plays a role in reactivating MAPK signaling after treatment with a targeted kinase inhibitor. We predict that STAG3 plays a role in ovarian cancer tumorigenesis, based on its gene fusions and upregulated expression, as well as the central role in cancer of the mitotic cohesin proteins STAG1 and STAG2, whose mutation or inactivation cause aneuploidy [94].

This study had several important limitations. First, the sample sizes in our initial SBT vs. HGSOC and stage II vs. stage III HGSOC comparisons were small. Nevertheless, we were able to validate the SBT vs. HGSOC panel through classification of an independent dataset containing 267 samples. Given the distinct differences in SBT and HGSOC, small sample sizes appear not to hinder differential biomarker identification. In contrast, the stage II vs. stage III HGSOC panel was not successful in distinguishing TCGA stage II vs. stage III tumors. In the future it may be useful to repeat this comparison using newer prognostic classifications for ovarian cancer based on machine learning, which have the potential for higher accuracy than FIGO staging [95]. Second, in our initial comparison of the HGSOC stages, we did not have access to RNA-seq from stage I samples, which are rarely identified, or stage IV samples. Stage IV samples were present in TCGA data, however, and helped confirm the differential expression of *GCLC* between different HGSOC stages. Third, the only ChIP-seq data from ovarian cancer cells that we obtained was from the PEO4 cell line. PEO4 expresses ERα and PITX1, but it remains unclear how characteristic it is of HGSOCs. In the future, it would be helpful to validate our TF findings in additional ovarian cancer cell lines. Fourth, our differential gene expression analysis did not employ multiple test correction due to the very small sample sizes.

This study also has important strengths. To the best of our knowledge, this is the first study to identify members of a transcriptional network, overlapping in content with the MegaTrans complex, that regulates differential gene expression in ovarian tumors. Of note, one of the TFs we identified as playing a role in invasiveness, PITX1, has not previously been implicated in ovarian cancer or the MegaTrans complex. It is surprising that, from such a small sample size, we were able to identify a host of regulatory factors shared among promoters of genes distinctive for different subtypes or stages of malignancy. Previous studies identify the role of MegaTrans in enhancers rather than promoters [22]; however, based on our data, binding motifs for these factors can also exist in DEG promoters.

In conclusion, the novel alterations associated with invasiveness in this study—including DEGs, novel fusion genes, and upstream regulatory elements—may help us learn more about the mechanisms responsible for malignancy in HGSOCs. Future molecular studies should explore and delineate the roles of FOXF1 and FOXA1 in SBTs and HGSOCs, as well as characterize PITX1 interactions with components of the MegaTrans complex. Future work could also attempt to identify which regulators bind the remaining binding sites upstream of DEGs that we were unable to match to TFs. For 14 different predicted binding site motifs, we identified PITX1, RARA, BHLHE41, MYC/MAX, and FOXF1 as candidate binding factors, and later added RUNX1 and TFAP2C. The remaining binding factors could comprise additional auxiliary members of a master regulatory complex. A more detailed understanding of the dynamic molecular alterations that occur during malignant disease may propel the field forward and create much-needed new avenues for EOC management.

## Supplementary Information

**Additional file 1.**

**Additional file 2.**

## Data Availability

The datasets used and/or analyzed during the current study will be made available through dbGAP, at NCBI Sequence Read Archive (RNA-seq files) and NCBI dbSNP, NCBI dbVar (.csv files). ChIP-seq data will be deposited into GEO.
