# Supplementary material for: Differential gene expression identifies a transcriptional regulatory network involving ER-alpha and PITX1 in invasive epithelial ovarian cancer
Source: BMC Cancer. 2021 Jul 3;21:768. doi: 10.1186/s12885-021-08276-8 (PMC8254236; doi:10.1186/s12885-021-08276-8)
Supplement: Supplementary file 1 — Additional file 1. [file 12885_2021_8276_MOESM1_ESM.zip › Yichao_supplemental_Tables_updated 032421.pdf]

Supplemental Tables for:

Differential gene expression identifies a transcriptional regulatory network involving ERα and PITX1 in invasive epithelial ovarian cancer

Yichao Li[1], Sushil Jaiswal[2], Rupleen Kaur[2], Dana Alsaadi[2], Xiaoyu Liang[1], Frank Drews[1], Julie A. DeLoia[3], Thomas Krivak[4], Hanna M. Petrykowska[2], Valer Gotea[2], Lonnie Welch[1], and Laura Elnitski[2]*

Table S1. Summary of RNA sequence alignment data from different ovarian tumor samples

Sample type	Stage	Number of tissue samples	Range of total RNA-seq reads per tissue type (millions)	Overall read alignment rate (%)	Concordant pair alignment rate (%)
High-grade serous ovarian cancer	II	3	20.0–31.5	78.5–94.3	61.9–83.8
	III	4	34.3–34.6	76.8–91.8	52.3–78.6
Serous borderline tumor	I	4	32.4–34.1	76.3–93.4	51.4–80.7

Table S2. Functional pathway analyses

GO group	GO identifier	Number of genes	P-value	Mean of GO group	Mean of non-group members
**Serous borderline tumors**					
Regulation of mature B cell apoptotic process	GO:0002905	4	1.14E-22	989.71	20.49
Negative regulation of mature B cell apoptotic process	GO:0002906	4	1.14E-22	989.71	20.49
Cytosolic large ribosomal subunit	GO:0022625	18	3.75E-15	403.59	20.22
Dopachrome isomerase activity	GO:0004167	3	4.07E-15	851.32	20.60
Large ribosomal subunit	GO:0015934	20	4.80E-14	372.12	20.22
Regulation of plasma membrane organization	GO:1903729	4	9.21E-13	687.03	20.59
Translational initiation	GO:0006413	27	7.79E-10	280.10	20.29
Sequestering of zinc ion	GO:0032119	4	5.93E-09	472.54	20.60
Regulation of leukocyte activation	GO:0002694	10	1.19E-08	360.95	20.51
Intramolecular oxidoreductase activity transposing C=C bonds	GO:0016863	6	1.23E-08	462.99	20.60
Chemokine production	GO:0032602	5	2.25E-08	439.10	20.60
**High-grade serous ovarian cancers, stage II**					
Tat protein binding	GO:0030957	4	4.53E-58	889.64	16.57
Nua4 histone acetyltransferase complex	GO:0035267	5	3.17E-46	673.05	16.55
Ribonucleoprotein granule	GO:0035770	8	1.19E-37	510.52	16.52
Histone methyltransferase complex	GO:0035097	8	1.99E-35	521.00	16.56
Nitric-oxide synthase binding	GO:0050998	7	2.60E-26	406.81	16.58
Substantia nigra development	GO:0021762	19	1.47E-25	238.39	16.31
Nucleosomal DNA binding	GO:0031492	10	6.52E-25	379.51	16.58
Nucleosome binding	GO:0031491	12	1.30E-21	328.02	16.58
Cell junction organization	GO:0034330	10	9.50E-19	286.38	16.58
Fc-gamma receptor signaling pathway involved in phagocytosis	GO:0038096	15	1.76E-18	262.23	16.55

**High-grade serous ovarian cancers, stage III**					
Sequestering of actin monomers	GO:0042989	3	6.32E-68	1164.00	15.47
Filamentous actin	GO:0031941	9	2.63E-19	333.87	15.49
Maintenance of protein location in cell	GO:0032507	12	2.17E-17	296.84	15.48
Actin monomer binding	GO:0003785	8	2.27E-16	279.55	15.48
Cellular component	GO:0005575	3173	5.78E-16	5.83	26.10
Membrane	GO:0016020	2583	1.74E-11	5.82	22.93
Intrinsic component of membrane	GO:0031224	1986	1.57E-10	4.20	21.38
Integral component of membrane	GO:0016021	1938	3.15E-10	4.21	21.20
Cell part	GO:0044464	1985	3.63E-08	5.70	20.56
Actin cytoskeleton organization	GO:0030036	42	5.47E-08	96.73	15.31
Plasma membrane	GO:0005886	1595	1.08E-07	4.57	19.80
Actin filament-based process	GO:0030029	31	5.10E-07	104.72	15.44
Regulation of leukocyte activation	GO:0002694	6	1.25E-06	219.19	15.70
Mitochondrial electron transport cytochrome c to oxygen	GO:0006123	8	2.66E-06	195.65	15.69
S100 protein binding	GO:0044548	3	4.10E-06	209.09	15.71
Regulation of plasma membrane organization	GO:1903729	3	4.37E-06	218.98	15.72

Table S3 Fusion genes detected in serous borderline tumors (SBTs)[a]

Gene 1	Gene 2	Gene 1 expression	Gene 2 expression	Fusion gene expression	EricScore
STAG3L5P-PVRIG2P-PILRB	STAG3	3.93	0.89	28.02	0.96
STAG3L5P-PVRIG2P-PILRB	STAG3	6.12	1.48	67.24	0.77
STAG3L5P-PVRIG2P-PILRB	STAG3	0.54	1.43	34.81	0.98
STAG3L5P-PVRIG2P-PILRB	STAG3	1.12	2.3	30.52	0.98
SFT2D2	TBX19	40.51	2.53	1.87	0.53
SFT2D2	EEF1A1	17.7	22.1	39.58	0.61
SFT2D2	ACTN1	19.43	68.4	55.22	0.59
SFT2D2	MIPOL1	20.93	3.57	19.64	0.96
SMG1	SLC7A5	14.83	18.18	4.4	0.94
SMG1	BOLA2B	14.83	0.82	69.63	0.93
SMG1	BOLA2	9.16	15.02	72.54	0.93
SMG1	SMCHD1	9.16	7.1	28.8	0.70
SMG1	BOLA2B	10.04	1.2	62.33	0.94
SMG1	BOLA2B	12.99	0.75	19.27	0.89
KPNA2	BPTF	11.6	38.93	74.73	0.92
KPNA2	BPTF	5.64	5.38	103.39	0.54
KPNA2	BPTF	5.99	28.66	77.91	0.87
KPNA2	BPTF	4.11	28.15	103.08	0.93
STAG3	STAG3L5P-PVRIG2P-PILRB	0.89	3.93	28.02	0.96
STAG3	AKR1B1	0.89	15.77	13.43	0.90
STAG3	SLC2A5	1.48	0.93	28.42	0.98
STAG3	STAG3L5P-PVRIG2P-PILRB	1.48	6.12	67.24	0.77
STAG3	STAG3L5P-PVRIG2P-PILRB	1.43	0.54	34.81	0.98
STAG3	STAG3L5P-PVRIG2P-PILRB	2.3	1.12	30.52	0.98
BPTF	KPNA2	38.93	11.6	74.73	0.92
BPTF	LRRC37A	5.38	0.17	18.89	0.83
BPTF	KPNA2	5.38	5.64	103.39	0.54
BPTF	KPNA2	28.66	5.99	77.91	0.87
BPTF	KPNA2	28.15	4.11	103.08	0.93
BPTF	NEK5	28.15	30.66	10.46	0.62

[a] Gene 1 column shows the common genes in all four SBT samples, column two names the partners, and columns three and four are expression levels of the individual genes.

Table S4. Fusion genes detected in high-grade serous ovarian cancers (HGSOC) stage II tumors[a]

Gene 1	Gene 2	Gene 1 expression	Gene 2 expression	Fusion gene expression	EricScore
HHLA1	HHLA3	1.72	10.31	14.17	0.91
HHLA1	NADSYN1	2.74	4.5	21.87	0.88
HHLA1	TIMM50	0.88	11.68	24.22	0.98
HHLA1	ABHD12B	0.88	1.4	30.98	0.97
HHLA1	LRRK1	0.88	3.59	46.3	0.91
EMC3	CIDEC	16.67	2.14	37.49	0.95
EMC3	CIDEC	12.4	1.62	20.99	0.98
EMC3	CIDEC	24.8	2.09	60.63	0.94
SPINT2	C19orf33	74.16	51.83	50.8	0.93
SPINT2	C19orf33	44.26	52.41	218.94	0.94
SPINT2	C19orf33	18.24	5.54	27.37	0.95
STAG3L5P-PVRIG2P-PILRB	STAG3	0.62	1.68	30.54	0.84
STAG3L5P-PVRIG2P-PILRB	STAG3	3.34	1.42	39.77	0.97
STAG3L5P-PVRIG2P-PILRB	STAG3	3.75	1.64	23.55	0.92
C19orf33	SPINT2	51.83	74.16	50.8	0.93
C19orf33	SPINT2	52.41	44.26	218.94	0.94
C19orf33	SPINT2	5.54	18.24	27.37	0.95
CIDEC	EMC3	2.14	16.67	37.49	0.95
CIDEC	EMC3	1.62	12.4	20.99	0.98
CIDEC	EMC3	2.09	24.8	60.63	0.94
SEPT14	FBXO25	3.98	3.69	9.61	0.83
SEPT14	FBXO25	3.04	2.15	3.77	0.84
SEPT14	FBXO25	4.17	6.29	16.56	0.93
CRISPLD2	CYB561A3	4.09	7.33	44.72	0.97
CRISPLD2	GTDC1	2.21	1.62	32.01	0.96
CRISPLD2	IFITM2	20.75	63.17	119.11	0.53
FBXO25	SEPT14	3.69	3.98	9.61	0.83
FBXO25	SEPT14	2.15	3.04	3.77	0.84
FBXO25	SEPT14	6.29	4.17	16.56	0.93
PRR11	SOX13	3.79	8.02	57.77	0.91
PRR11	TECR	3.79	12.11	18.81	0.88
PRR11	SOX13	8.26	5.22	53.09	0.94
PRR11	SLC25A16	22.38	9.24	32.28	0.98
PRR11	SOX13	22.38	2.38	33.56	0.90
SOX13	PRR11	8.02	3.79	57.77	0.91
SOX13	PRR11	5.22	8.26	53.09	0.94
SOX13	PRR11	2.38	22.38	33.56	0.90
KPNA2	BPTF	6.09	13.2	54.81	0.93
KPNA2	BPTF	8.51	16.3	56.52	0.80
KPNA2	BPTF	16.05	27.02	77.13	0.88

STAG3	STAG3L5P-PVRIG2P-PILRB	1.68	0.62	30.54	0.84
STAG3	STAG3L5P-PVRIG2P-PILRB	1.42	3.34	39.77	0.97
STAG3	STAG3L5P-PVRIG2P-PILRB	1.64	3.75	23.55	0.92
BPTF	KPNA2	13.2	6.09	54.81	0.93
BPTF	KPNA2	16.3	8.51	56.52	0.80
BPTF	KPNA2	27.02	16.05	77.13	0.88
BCL7C	SETMAR	10.6	10.17	26.83	0.94
BCL7C	PGM3	14.82	5.33	17.74	0.83
BCL7C	PGM3	10.54	9.13	11.69	0.68
HHLA1	HHLA3	1.72	10.31	14.17	0.91
HHLA1	NADSYN1	2.74	4.5	21.87	0.88
HHLA1	TIMM50	0.88	11.68	24.22	0.98
HHLA1	ABHD12B	0.88	1.4	30.98	0.97
HHLA1	LRRK1	0.88	3.59	46.3	0.91

[a] Gene 1 column shows the common genes in all three HGSOC stage II samples, column two names the partners, and columns three and four are expression levels of the individual genes.

Table S5. Fusion genes detected in high-grade serous ovarian cancers (HGSOC) stage III tumors[a]

Gene 1	Gene 2	Gene 1 expression	Gene 2 expression	Fusion gene expression	EricScore
TMEM165	POLN	9.98	2.6	22.38	0.95
TMEM165	NDUFB2	12.11	12.72	28.7	0.96
TMEM165	WDR66	6.94	3.17	47.79	0.89
TMEM165	RNF220	6.94	2.11	6.09	0.58
TMEM165	RNF220	16.4	4.69	23.84	0.65
TBC1D17	TMEM181	17.19	6.46	73	0.89
TBC1D17	TP53	20.66	26.11	42.39	0.93
TBC1D17	NPFFR1	6.83	4.69	29.48	0.98
TBC1D17	NOM1	9.63	5.45	16.36	0.72
TBC1D17	TMEM181	9.63	8.52	43.71	0.55
DNTTIP2	ASB3	15.84	25.92	34.05	0.95
DNTTIP2	FRK	15.84	39.87	148.68	0.95
DNTTIP2	ZNF480	15.84	4.17	10.49	0.67
DNTTIP2	ZNF200	18.77	6.02	19.16	0.93
DNTTIP2	MIPOL1	10.45	24.34	72.55	0.80
DNTTIP2	KCNE4	17.54	1.83	20.6	0.51
RASEF	CCDC90B	7.52	15.02	7.78	0.97
RASEF	BCAS3	10.8	2.07	3.77	0.77
RASEF	CCDC90B	9.76	3.49	15.77	0.79
RASEF	STK3	9.76	4.8	12.31	0.50
RASEF	MRPS5	12.55	20.33	152.24	0.94
RASEF	STK3	12.55	7.89	49.25	0.78
ZNF207	CASC4	9.96	13.56	25.01	0.67
ZNF207	LCMT2	13.74	2.49	18.33	0.97
ZNF207	PPP2R5E	13.74	11.57	28.76	0.80
ZNF207	TMEM33	13.74	6.47	14.57	0.68
ZNF207	LCMT2	7.24	1.63	17.37	0.75
ZNF207	LCMT2	11.07	3.14	18.75	0.77
KCNK6	WDR97	14.39	4.07	14.67	0.93
KCNK6	IL4R	10.46	4.82	43.05	0.82
KCNK6	SSTR2	7.48	4.12	37.34	0.97
KCNK6	IL4R	7.48	4.2	6.84	0.92
KCNK6	AFF4	7.48	5.46	28.25	0.59
KCNK6	AFF4	9.68	12.37	26.66	0.92
MRPS22	SFPQ	3.47	35.67	57.1	0.94
MRPS22	COPB2	3.73	24.56	35.61	0.54
MRPS22	SFPQ	2.03	10.64	31.73	0.91
MRPS22	COPB2	1.61	21.62	5.19	0.90
MRPS22	SFPQ	1.61	46.83	54.61	0.88
STK33	SPEF2	12.07	7.58	16.59	0.81

STK33	RSRP1	4.13	44.25	61.67	0.97
STK33	GYPE	3.7	0.46	17.88	0.69
STK33	GADL1	3.57	4.32	18.69	0.61
STAG3	STAG3L5P-PVRIG2P-PILRB	3.49	2.54	55.91	0.91
STAG3	PILRB	2.54	0.53	10.81	0.90
STAG3	RFC1	3.49	10.79	14.41	0.79
STAG3	STAG3L5P-PVRIG2P-PILRB	2.59	8.21	58.14	0.91
STAG3	RFC1	0.57	8.68	19.62	0.71
STAG3	STAG3L5P-PVRIG2P-PILRB	0.53	2.82	31.77	0.96
ZNF480	PUS7L	4.17	6.24	14.78	0.71
ZNF480	DNTTIP2	4.17	15.84	10.49	0.67
ZNF480	DDHD1	12.06	2.65	102	0.95
ZNF480	FRK	12.06	45.31	31.78	0.73
ZNF480	CYP46A1	12.06	13.27	336.09	0.61
ZNF480	ZBTB20	24.69	8.97	26.82	0.55
ZNF480	TMEM212	7.03	14.07	31.41	0.88
ZNF480	SP100	7.03	11.18	31.7	0.70
ZNF480	SP100	7.03	11.18	49.27	0.61
LCMT2	DARS	2.99	21.99	37.98	0.62
LCMT2	ZNF207	2.49	13.74	18.33	0.97
LCMT2	ZNF207	1.63	7.24	17.37	0.75
LCMT2	ZNF207	3.14	11.07	18.75	0.77
FCRL5	TPRXL	0.76	2.79	479.25	0.69
FCRL5	PPP1CB	1.54	45.51	19.98	0.96
FCRL5	EFHC1	1.54	9.68	73.86	0.91
FCRL5	CCDC122	1.54	3.76	301.72	0.58
FCRL5	ADAM32	1.54	0.53	86.64	0.51
FCRL5	EFHC1	0.84	6.44	298.15	0.71
FCRL5	TPRXL	0.84	2.31	147.83	0.52
FCRL5	CCDC122	0.3	7.26	270.32	0.62
TMEM165	POLN	9.98	2.6	22.38	0.95
TMEM165	NDUFB2	12.11	12.72	28.7	0.96
TMEM165	WDR66	6.94	3.17	47.79	0.89
TMEM165	RNF220	6.94	2.11	6.09	0.58
TMEM165	RNF220	16.4	4.69	23.84	0.65

[a] Gene 1 column shows the common genes in all four HGSOC stage III samples, column two names the partners, and columns three and four are expression levels of the individual genes.

Table S6. Expression of target genes in a validation dataset (GSE9891) of 18 serous borderline tumors (SBTs) vs. 247 high-grade serous ovarian cancers (HGSOCs)

		SBT expression		HGSOC expression		[$]SBT/ HGSOC	
**Gene**	**Probe**	**Mean**	**Std Dev**	**Mean**	**Std Dev**	**Fold Change**	**P-value**
SLC7A2 [a]	225516_at	2214.42	996.62	339.65	552.05	6.52	2.47E-14
PIFO	228100_at	862.10	518.90	166.09	233.09	5.19	1.66E-08
BBS12	229603_at	112.03	29.50	44.47	27.10	2.52	1.12E-12
HES2 [a]	231928_at	155.88	59.30	75.84	30.53	2.06	2.09E-06
RPL7A [a]	224930_x_at	14162.08	1219.72	10756.23	2520.77	1.32	1.71E-14
AFF2 [a]	206105_at	21.11	9.54	16.46	6.01	1.28	2.99E-02
RPL12 [a]	200809_x_at	15593.41	805.01	12176.07	2366.20	1.28	6.96E-22
RPL12 [a]	214271_x_at	9809.73	489.16	7752.93	1591.69	1.27	3.75E-22
RPL12 [a]	200088_x_at	16151.42	790.57	12764.32	2327.47	1.27	7.12E-22
RPL7A [a]	217740_x_at	15559.66	858.99	12328.97	2460.68	1.26	4.48E-20
RPS12	213377_x_at	13803.33	831.12	11606.89	2119.25	1.19	1.43E-13
RPS15	200819_s_at	12224.12	662.68	10522.00	2103.88	1.16	5.55E-13
HES2 [a]	214521_at	15.70	3.05	14.19	2.75	1.11	4.96E-02
HES2 [a]	216674_at	12.96	2.51	11.69	2.09	1.11	4.56E-02
SLC7A2 [a]	207626_s_at	11.21	2.81	10.36	3.61	1.08	1.15E-01
AFF2 [a]	210957_s_at	15.32	3.18	14.95	2.53	1.02	6.67E-01
AFF2 [a]	216364_s_at	17.33	2.55	17.58	3.70	-1.01	8.56E-01
MAFB [a]	222670_s_at	154.60	40.50	490.14	291.08	-3.17	1.20E-14
MAFB [a]	218559_s_at	240.78	88.08	962.61	638.42	-4.00	1.36E-12
CRABP2	202575_at	292.42	226.26	1758.05	1277.94	-6.01	1.36E-09

[$]For any value smaller than 1 (i.e. for downregulation), the fold change value was replaced by its negative reciprocal value. [a] Multiple probes per gene. Data were collected on a Affymetrix microarray.

Table S7. Putative promoter elements of differentially expressed genes in serous borderline tumor (SBT) vs. high-grade serous ovarian cancer (HGSOC) samples[a]

Motif Logo	Motif Name	%Fore	#Fore	%Back	#Back	Accuracy
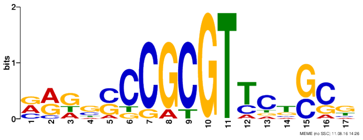	Improbizer_GAGGCCCGCGTTCTGCG	100%	9	19.4%	7	84.4%
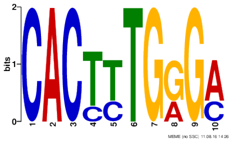	DME_CACYYTGRGM	100%	9	33.3%	12	73.3%
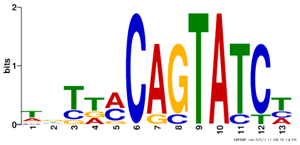	Improbizer_TGTTACAGTATCT	88.9%	8	13.9%	5	86.6%
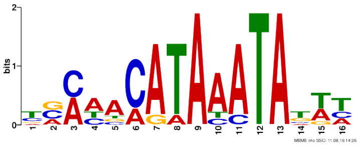	Improbizer_TCCAACATAAATATTT	88.9%	8	16.7%	6	84.4%
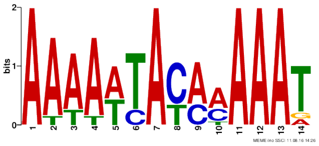	AAAAWTAYMMAAAT_14	88.9%	8	25%	9	77.7%
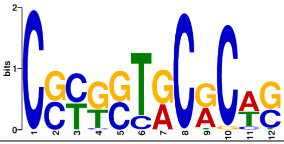	Homer_12_5	88.9%	8	33.3%	12	71.1%

[a] %Fore: foreground promoter coverage; #Fore: the number of foreground gene promoters covered by the given motif; %Back: background promoter coverage; #Back: the number of background gene promoters covered by the given motif.

Table S8. Putative promoter motifs of differentially expressed genes in high-grade serous ovarian cancer (HGSOC) stage II vs. stage III tumors[a]

Motif Logo	Motif Name	%Fore ground	#Fore ground	%Back ground	#Back ground	Accuracy
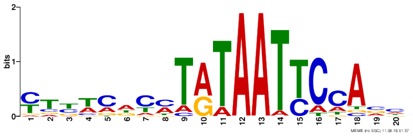	Improbizer_CTTTCACCTATAATTCCACC	94.1	16	32.4	22	72.9%
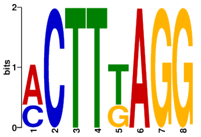	DME_MCTTKAGG	64.7	11	11.8	8	83.5%
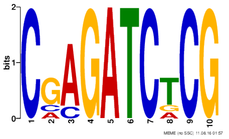	Weeder_2	64.7	11	23.5	16	74.1%
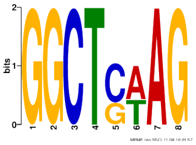	DME_GGCTSWAG	64.7	11	27.9	19	70.6%
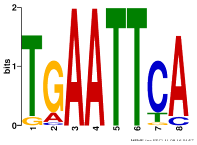	DECOD_Motif2_8	52.9	9	16.2	11	77.6%
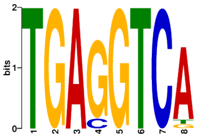	DME_TGAGGTCW	52.9	9	17.6	12	76.5%
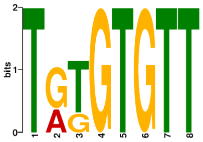	DME_TRKGTGTT	52.9	9	17.6	12	76.5%
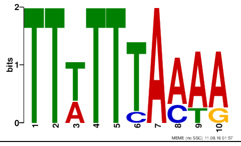	DME_TTWTTYAMWR	52.9	9	25	17	70.6%

[a] %Fore: foreground promoter coverage; #Fore: the number of foreground gene promoters covered by the given motif; %Back: background promoter coverage; #Back: the number of background gene promoters covered by the given motif.

Table S9: See separate excel file for UNIBIND ChIP-seq data for the 28 DEG promoters

Table S10. Cancer associations of 28 differentially expressed genes (DEGs) identified in serous borderline tumor (SBT) vs high-grade serous ovarian cancer (HGSOC) and stage II vs stage III HGSOC comparisons

HGSOC-stage 2 vs HGSOC-stage 3 DEGs	Ovarian cancer (OC)	Previous cancer association or function	SBT vs HGSOC DEGs	Ovarian cancer (OC)	Previous cancer association or function
TSPAN1[1]	Known	Stage III serous ovarian carcinomas	*SLC7A2 [2]*	Known	Transporter protein; prognostic model for OC
CLIC1 [3]	Known	Biomarker of intraperitoneal metastasis in ovarian cancer	*PIFO [4] [5]*	Known	Part of Hedgehog signaling pathway, which has key role in OC
NQO1 [6]	Known	Poor prognosis of serous ovarian tumors	*RPL7A [7]*	Known	Found within OC susceptibility loci
DNAJB9 [8]	Known	Related to serous ovarian carcinomas	*CRABP2 [9]*	Known	Retinoic acid binding protein specifically expressed in ovarian serous carcinoma
CHST15 [10]	Unknown	Astrocytic tumors	*BBS12*	Unknown	Related to Bardet-Biedl Syndrome; genetic disease that can involve hypoplasia of uterus, ovaries, and fallopian tubes.
ECE1 [11]	Unknown	Prostate cancer	*HES2*	Unknown	Part of gene family that represses tissue specific transcription; associated with colorectal cancer
PGK1 [12]	Unknown	Breast and colon cancer	*RPS12, RPL12, RPS15*	Unknown	Encode ribosomal proteins implicated as having a role in oncologic diseases
CA12 [10]	Unknown	Renal and lung cancer	*MAFB*	Unknown	Myeloma
PPP1R14C [13]	Unknown	Breast, pancreatic, lung, and gastric cancers	*AFF2*	Unknown	Mutated forms associated with breast cancer
ANK1 [14]	Unknown	Breast, pancreatic, lung, and gastric cancers			
GCLC [15]	Unknown	Breast, pancreatic, lung, and gastric cancers			
SERPINE2 [16]	Unknown	Breast, pancreatic, lung, and gastric cancers			
ZDHHC7	Unknown	No previous published association with cancer			
ENPP4	Unknown				
TMEM30B	Unknown				
SYNPO	Unknown				
PDGFC	Unknown				

1. Scholz CJ, Kurzeder C, Koretz K, Windisch J, Kreienberg R, Sauer G, Deissler H: **Tspan-1 is a tetraspanin preferentially expressed by mucinous and endometrioid subtypes of human ovarian carcinomas.** *Cancer Lett* 2009, **275:**198-203.
2. Sabatier R, Finetti P, Bonensea J, Jacquemier J, Adelaide J, Lambaudie E, Viens P, Birnbaum D, Bertucci F: **A seven-gene prognostic model for platinum-treated ovarian carcinomas.** *Br J Cancer* 2011, **105:**304-311.
3. Ye Y, Yin M, Huang B, Wang Y, Li X, Lou G: **CLIC1 a novel biomarker of intraperitoneal metastasis in serous epithelial ovarian cancer.** *Tumour Biol* 2015, **36:**4175-4179.
4. Jung B, Messias AC, Schorpp K, Geerlof A, Schneider G, Saur D, Hadian K, Sattler M, Wanker EE, Hasenöder S, Lickert H: **Novel small molecules targeting ciliary transport of Smoothened and oncogenic Hedgehog pathway activation.** *Sci Rep* 2016, **6:**22540.
5. Szkandera J, Kiesslich T, Haybaeck J, Gerger A, Pichler M: **Hedgehog signaling pathway in ovarian cancer.** *Int J Mol Sci* 2013, **14:**1179-1196.
6. Cui X, Li L, Yan G, Meng K, Lin Z, Nan Y, Jin G, Li C: **High expression of NQO1 is associated with poor prognosis in serous ovarian carcinoma.** *BMC Cancer* 2015, **15:**244.
7. Kuchenbaecker KB, Ramus SJ, Tyrer J, Lee A, Shen HC, Beesley J, Lawrenson K, McGuffog L, Healey S, Lee JM, et al: **Identification of six new susceptibility loci for invasive epithelial ovarian cancer.** *Nat Genet* 2015, **47:**164-171.
8. Lee HJ, Kim JM, Kim KH, Heo JI, Kwak SJ, Han JA: **Genotoxic stress/p53-induced DNAJB9 inhibits the pro-apoptotic function of p53.** *Cell Death Differ* 2015, **22:**86-95.
9. Toyama A, Suzuki A, Shimada T, Aoki C, Aoki Y, Umino Y, Nakamura Y, Aoki D, Sato TA: **Proteomic characterization of ovarian cancers identifying annexin-A4, phosphoserine aminotransferase, cellular retinoic acid-binding protein 2, and serpin B5 as histology-specific biomarkers.** *Cancer Sci* 2012, **103:**747-755.
10. Kobayashi T, Yan H, Kurahashi Y, Ito Y, Maeda H, Tada T, Hongo K, Nakayama J: **Role of GalNAc4S-6ST in astrocytic tumor progression.** *PLoS One* 2013, **8:**e54278.
11. Whyteside AR, Hinsley EE, Lambert LA, McDermott PJ, Turner AJ: **ECE-1 influences prostate cancer cell invasion via ET-1-mediated FAK phosphorylation and ET-1-independent mechanisms.** *Can J Physiol Pharmacol* 2010, **88:**850-854.
12. Sun S, Liang X, Zhang X, Liu T, Shi Q, Song Y, Jiang Y, Wu H, Jiang Y, Lu X, Pang D: **Phosphoglycerate kinase-1 is a predictor of poor survival and a novel prognostic biomarker of chemoresistance to paclitaxel treatment in breast cancer.** *Br J Cancer* 2015, **112:**1332-1339.
13. Scanlan MJ, Gout I, Gordon CM, Williamson B, Stockert E, Gure AO, Jäger D, Chen YT, Mackay A, O'Hare MJ, Old LJ: **Humoral immunity to human breast cancer: antigen definition and quantitative analysis of mRNA expression.** *Cancer Immun* 2001, **1:**4.
14. Omura N, Mizuma M, MacGregor A, Hong SM, Ayars M, Almario JA, Borges M, Kanda M, Li A, Vincent A, et al: **Overexpression of ankyrin1 promotes pancreatic cancer cell growth.** *Oncotarget* 2016, **7:**34977-34987.
15. Nichenametla SN, Muscat JE, Liao JG, Lazarus P, Richie JP, Jr.: **A functional trinucleotide repeat polymorphism in the 5'-untranslated region of the glutathione biosynthetic gene GCLC is associated with increased risk for lung and aerodigestive tract cancers.** *Mol Carcinog* 2013, **52:**791-799.
16. Wang K, Wang B, Xing AY, Xu KS, Li GX, Yu ZH: **Prognostic significance of SERPINE2 in gastric cancer and its biological function in SGC7901 cells.** *J Cancer Res Clin Oncol* 2015, **141:**805-812.
