# Supplementary material for: Differential gene expression identifies a transcriptional regulatory network involving ER-alpha and PITX1 in invasive epithelial ovarian cancer
Source: BMC Cancer. 2021 Jul 3;21:768. doi: 10.1186/s12885-021-08276-8 (PMC8254236; doi:10.1186/s12885-021-08276-8)
Supplement: Supplementary file 2 — Additional file 2. [file 12885_2021_8276_MOESM2_ESM.pdf]

Supplemental Figures for:

Differential gene expression identifies a transcriptional regulatory network involving ERα and PITX1 in invasive epithelial ovarian cancer

Yichao Li[1], Sushil Jaiswal[2], Rupleen Kaur[2], Dana Alsaadi[2], Xiaoyu Liang[1], Frank Drews[1], Julie A. DeLoia[3], Thomas Krivak[4], Hanna M. Petrykowska[2], Valer Gotea[2], Lonnie Welch[1], and Laura Elnitski[2]*

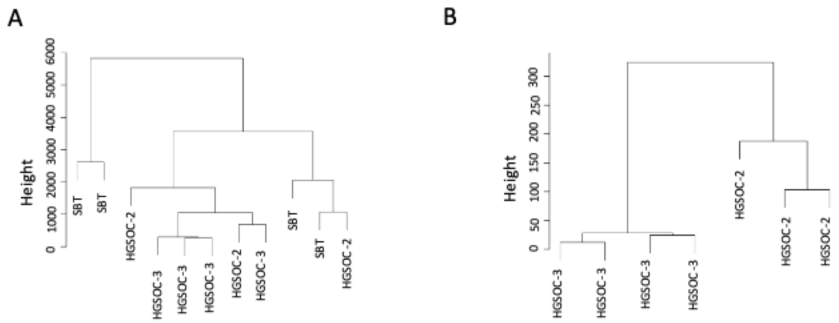

Figure S1. Hierarchical clustering of the SBT and HGSOC samples using the same gene expression data as in Figure 1. (A) Clustering of the SBT and HGSOC samples. (B) Clustering of the HGSOC stage II (HGSOC-2) and III (HGSOC-3) samples.

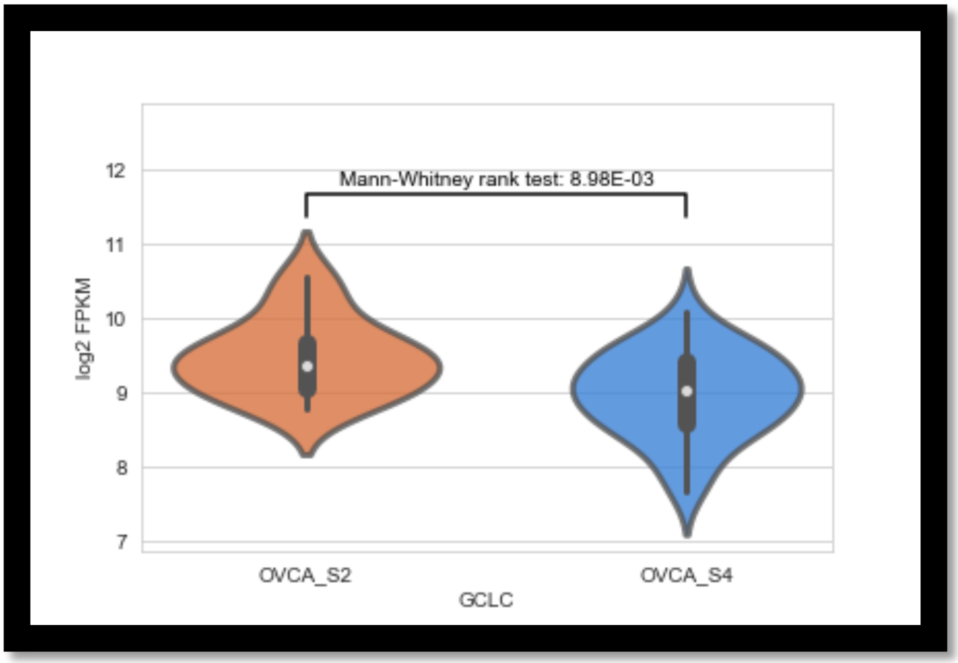

Figure S2. Differential gene expression of *GCLC* (*p*-value = 8.98 E-3) in stage II (OVCA_S2) and stage IV (OVCA_S4) high-grade serous ovarian cancers. Data were obtained from the TCGA ovarian cancer cohort. N=13 stage II and n=32 stage IV.

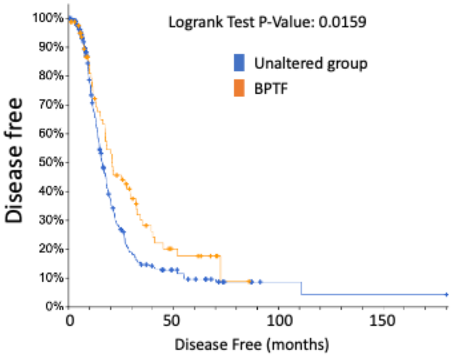

	# cases, total	# cases, deceased	median months disease free
Unaltered group	315	229	16.00
BPTF	81	54	20.83

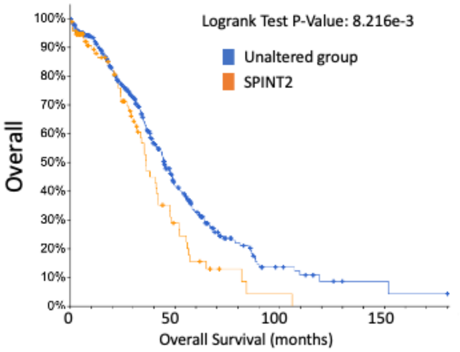

	# cases, total	# cases, deceased	median months overall
Unaltered group	393	219	44.81
SPINT2	92	53	36.24

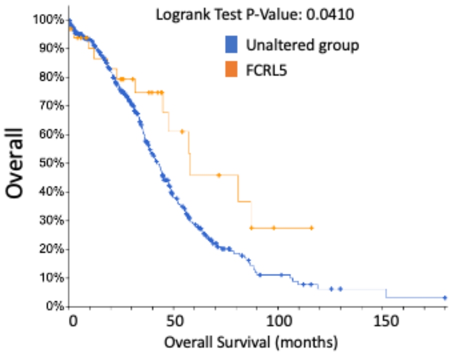

	# cases, total	# cases, deceased	median months overall
Unaltered group	453	259	42.02
FCRL5	32	13	58.05

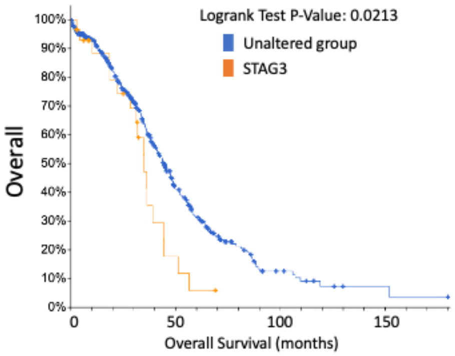

	# cases, total	# cases, deceased	median months overall
Unaltered group	456	254	44.45
STAG3	29	18	34.79

Figure S3. Association between survival and fusion genes identified in serous borderline tumors (SBT) or high-grade serous ovarian cancers (HGSOC) and applied to 563 patients with HGSOC. *BPTF* was involved in fusions in SBT and HGSOC stage II, *SPINT2* was involved in fusions in HGSOC stage II, *FCRL5* was involved in fusions in HGSOC stage III, *STAG3* was involved in fusions in all 11 tumors.

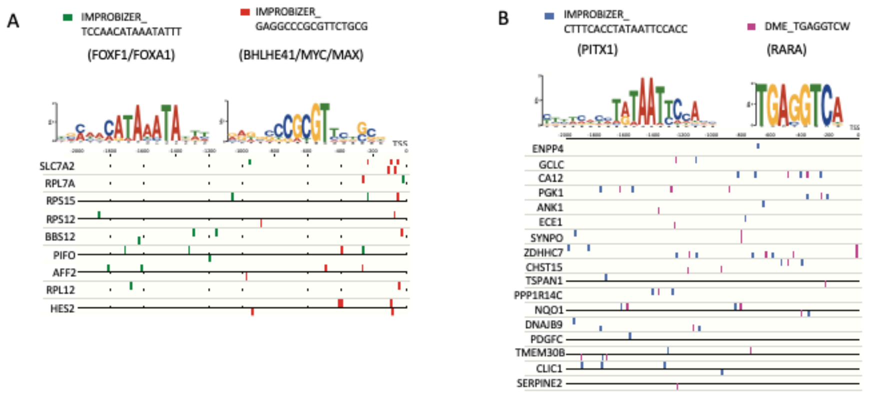

Figure S4. Transcription factors (TFs) implicated in the differential expression of genes associated with invasiveness in epithelial ovarian cancer. Motif enrichment in the promoter sequences of the differentially expressed genes we identified in our (A) serous borderline tumor (SBT) vs high-grade serous ovarian cancer (HGSOC) comparison, where *CRABP2* and *MAFB* were excluded, and (B) stage II vs. stage III HGSOC comparison. Top and bottom strand motifs are drawn above and below the horizontal line for each promoter region.
